# Dual roles of R-loops in the formation and processing of programmed DNA double-strand breaks during meiosis

**DOI:** 10.1186/s13578-023-01026-2

**Published:** 2023-05-11

**Authors:** Chao Liu, Wei Xu, Liying Wang, Zhuo Yang, Kuan Li, Jun Hu, Yinghong Chen, Ruidan Zhang, Sai Xiao, Wenwen Liu, Huafang Wei, Jia-Yu Chen, Qianwen Sun, Wei Li

**Affiliations:** 1 Guangzhou Women and Children’s Medical Center, Guangzhou Medical University, Guangzhou, 510623 China; 2 Center for Plant Biology, School of Life Sciences, Tsinghua University, Beijing, 100084 China; 3 State Key Laboratory of Pharmaceutical Biotechnology, School of Life Sciences, Chemistry and Biomedicine Innovation Center (ChemBIC), Nanjing University, Nanjing, 210023 China; 4 State Key Laboratory of Stem Cell and Reproductive Biology, Institute of Zoology, Stem Cell and Regenerative Medicine Innovation Institute, Chinese Academy of Sciences, Beijing, 100101 China; 5 Tsinghua-Peking Center for Life Sciences, Beijing, 100084 China; 6 University of Chinese Academy of Sciences, Beijing, 100049 China; 7 Shenzhen Branch, Guangdong Laboratory for Lingnan Modern Agriculture, Genome Analysis Laboratory of the Ministry of Agriculture, Agricultural Genomics Institute at Shenzhen, Chinese Academy of Agricultural Sciences, Shenzhen, 518120 China

**Keywords:** Meiosis, Meiotic recombination, Meiotic DSB hotspots, R-loops, Transcription-replication head-on collisions

## Abstract

**Background:**

Meiotic recombination is initiated by Spo11-dependent programmed DNA double-strand breaks (DSBs) that are preferentially concentrated within genomic regions called hotspots; however, the factor(s) that specify the positions of meiotic DSB hotspots remain unclear.

**Results:**

Here, we examined the frequency and distribution of R-loops, a type of functional chromatin structure comprising single-stranded DNA and a DNA:RNA hybrid, during budding yeast meiosis and found that the R-loops were changed dramatically throughout meiosis. We detected the formation of multiple *de novo* R-loops in the pachytene stage and found that these R-loops were associated with meiotic recombination during yeast meiosis. We show that transcription-replication head-on collisions could promote R-loop formation during meiotic DNA replication, and these R-loops are associated with Spo11. Furthermore, meiotic recombination hotspots can be eliminated by reversing the direction of transcription or replication, and reversing both of these directions can reconstitute the hotspots.

**Conclusions:**

Our study reveals that R-loops may play dual roles in meiotic recombination. In addition to participation in meiotic DSB processing, some meiotic DSB hotspots may be originated from the transcription-replication head-on collisions during meiotic DNA replication.

**Supplementary Information:**

The online version contains supplementary material available at 10.1186/s13578-023-01026-2.

## Background

Meiosis is a specialized type of cell division, during which DNA undergoes a single round of replication, followed by two consecutive cell divisions [1]. Prolonged prophase I is a remarkable feature of meiosis, including leptotene, zygotene, pachytene, diplotene and diakinesis, and a series of events occur during this process, such as meiotic DNA replication, the generation of programmed DNA double-strand breaks (DSBs), meiotic recombination, crossover formation, and synapsis [1, 2]. Meiotic recombination is indispensable for the exchange of genetic information and proper homologous chromosome segregation during meiosis, and this process is initiated through programmed DSBs, which is mediated by an evolutionarily conserved transesterase-like enzyme, Spo11 [3, 4]. Following meiotic DSB formation, the Mre11–Rad50–Xrs2 (MRX) complex plus Sae2 generates nicks on Spo11-bound strands to facilitate the DNA ends undergoing resection through modest 3′ to 5′ Mre11 exonuclease activity and robust 5′ to 3′ Exo1 exonuclease activity [5–7]. DNA resection generates 3′ single-stranded DNA (ssDNA) tails that serve as substrates for the recombinases DMC1 and RAD51 as they search for homology and invade a homologous repair template [8, 9]. The distribution of meiotic DSBs across the genome is not random; rather, it is preferentially concentrated within discrete, scattered, and permissive regions known as recombination hotspots [10–12]. Previous studies have detected correlations between these hotspots and histone modifications, transcription factors (TFs), and the meiotic chromatid cohesin complex [7, 10, 11, 13–15]. Although hotspots are thought to be determined by chromatin structure(s) [13], it remains unclear which types of chromatin structure contribute to hotspot formation.

A type of chromatin structure known as an R-loop comprises a DNA:RNA hybrid as well as its associated nontemplate single-stranded DNA [16, 17]. Accumulating evidence indicates that R-loops play dual roles in genome stability; on the one hand, R-loops accumulate *in cis* to DSBs and participate in DSB repair [18–20]. On the other hand, persistent R-loops result in genome instability, primarily by interfering with DNA replication [17, 21]. The collision of DNA replication forks with R-loops has been shown to cause fork stalling, which in turn generates DSBs and induces hyperrecombination by creating a damage-prone site on the genome [17, 22, 23]. During meiosis, programmed DSBs have also been functionally linked to premeiotic DNA replication, as blocking meiotic replication prevented meiotic DSB formation and recombination initiation [24, 25]. DNA:RNA hybrids have been reported to be formed at ssDNA ends of meiotic DSB sites and participate in meiotic recombination [26]. However, whether R-loops promote DSB formation during meiosis remains largely unknown.

Here, we found that R-loop accumulation perturbed genomic stability during budding yeast meiosis. Further detection of genome-wide R-loops by using ssDRIP-seq (single-strand DNA ligation-based library construction from DNA:RNA hybrid immunoprecipitation, followed by sequencing) during yeast meiosis showed obvious dynamic changes throughout meiosis. Multiple *de novo* R-loops at the pachytene stage were found to be associated with meiotic DSB hotspots during meiosis. We further revealed that transcription-replication head-on collisions during meiotic DNA replication could promote R-loop formation. The disruption of R-loop in Spo11 deletion strain showed stronger R-loop signals at the transcription-replication head-on collision regions compared to wild-type cells, which was similar to the *rnh1*/*rnh201Δ* strain, suggesting that meiotic DSBs may also promote the elimination of R-loops at the transcription-replication head-on collisions. Furthermore, some meiotic DSB hotspots can be eliminated by reversing the direction of either transcription or replication and reconstituted by reversing both of their directions. Therefore, R-loops may play dual roles during meiosis; in addition to participation in meiotic DSB processing, some meiotic DSB hotspots may originate from transcription-replication head-on collisions during meiotic DNA replication.

## Results

### Persistent R-loops perturbed genomic stability during meiosis

**Fig. 1:**
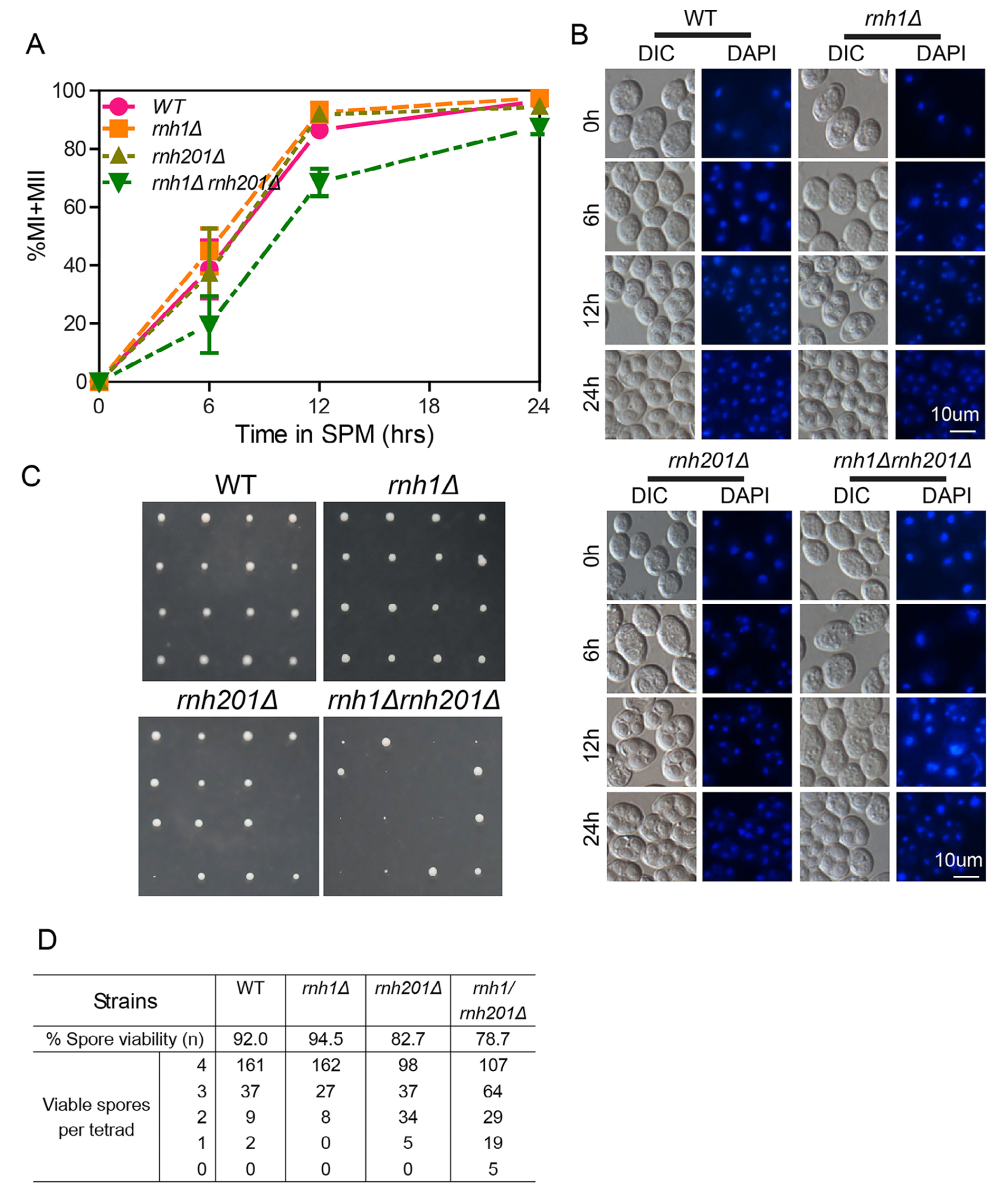
Lack of *RNH1/RNH201* perturbs genome stability during meiosis. (**A**) Disrupting *RNH1* and *RNH201* impaired meiotic progression. The percentage of nuclei that completed meiosis. Meiosis was induced in WT, *rnh1Δ*, *rnh201Δ* and *rnh1Δ/rnh201Δ* cells by transfer the cells to sporulation medium (SPM), and the cells were analysed at different time points (0 h, 6 h, 12 h, 24 h). (**B**) WT, *rnh1 Δ*, *rnh201Δ* and *rnh1/rnh201Δ* spores were stained with DAPI at different time points during sporulation (0 h, 6 h, 12 h, 24 h). (**C**) Spore viability in WT, *rnh1Δ*, *rnh201Δ*, and *rnh1/rnh201Δ *cells. The cells were induced to sporulate, and spore viability was monitored via dissection of tetrads from SPM. (**D**) The spore viability of WT, *rnh1 Δ*, *rnh201Δ* and *rnh1/rnh201Δ*cells.

To explore whether R-loops are involved in meiosis and to assess their potential relationship with DSB hotspots, we deleted the *RNH1* and *RNH201* genes in budding yeast; these genes encode RNase H enzymes that have been shown to specifically degrade the RNA within DNA:RNA hybrids, thereby eliminating R-loops [27–29]. Phenotypic observations revealed sporulation delays for the *rnh1/rnh201Δ* double deletion cells at 6 and 12 h after transfer to sporulation medium (Fig. 1A, B), which is similar to a recent report [26]. Furthermore, most of the tetrad spores generated by *rnh1/rnh201Δ* double mutant cells showed growth defects (Fig. 1C, D). These findings support that R-loop accumulation may be related to perturbed genomic stability and/or meiotic recombination.

### Genome-wide detection of R-loops in yeast by ssDRIP-seq

**Fig. 2:**
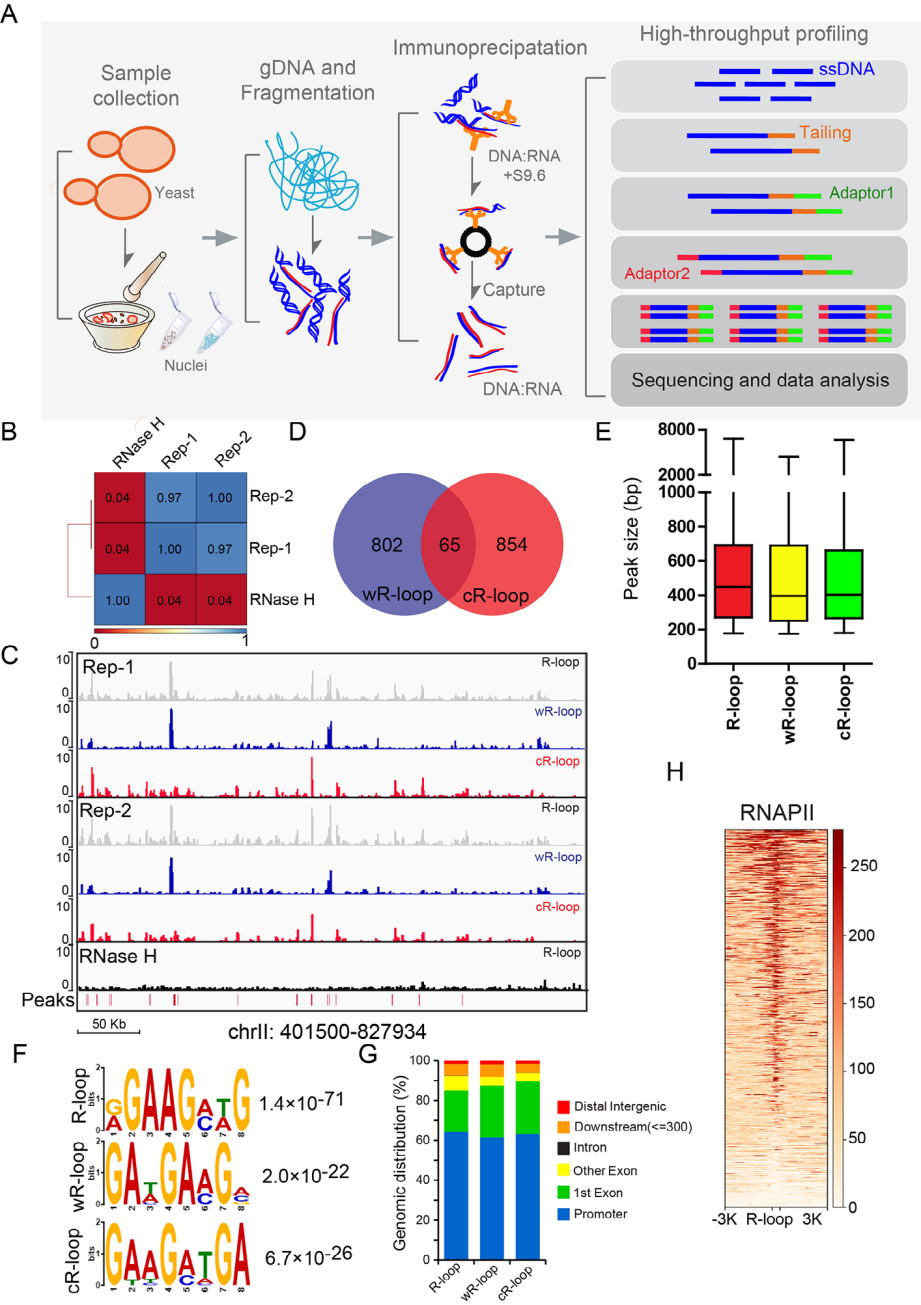
Genome-wide detection of R-loops in yeast by ssDRIP-seq. (**A**) Diagram of ssDRIP-seq in yeast under vegetative growth conditions. Nuclei were isolated from yeast cells by grinding well in liquid nitrogen, followed by genome DNA (gDNA) extraction. gDNA was fragmented using endonucleases, and DNA:RNA hybrids were captured by using the S9.6 antibody. The DRIPed DNA samples were ligated to the adapter on the 3’ end of the ssDNA using Adaptase. The extension step was performed using the primer paired to the first adapter, followed by a ligation reaction to add the second truncated adapter. An indexing PCR step was performed to add the indexed sequence, and the library was amplified and sequenced. (**B**) Pairwise comparison of two ssDRIP-seq replicates in mitosis. The Pearson correlation coefficient was computed from each comparison to evaluate the reproducibility. RNase H treatment served as a negative control. (**C**) Snapshot of the ssDRIP-seq data in yeast under vegetative growth conditions and RNase H treatment, including R-loop (grey), wR-loop (blue) and cR-loop (red) in a representative genomic region (chrII:401,500–827,934). (**D**) Overlap of peaks identified by wR-loop and cR-loop based on peak count in yeast under vegetative growth conditions. (**E**) The size distribution of R-loop (red), wR-loop (yellow) and cR-loop (green) peaks in yeast under vegetative growth conditions determined by the peak calling strategies of MACS2 with default settings to call narrow R-loop peaks [63]. (**F**) DNA motif in the peak regions of unstranded R-loops, wR-loops and cR-loops in yeast under vegetative growth conditions that were identified by MEME-ChIP. E-values are provided on the right. (**G**) The genomic distribution of ssDRIP-seq mapped R-loops, wR-loops and cR-loops that were identified by ChIPseeker. Various genomic regions are colour coded according to the labels on the bottom. Blue, promoter; green, 1st exon; yellow, other exon; black, intron; orange, downstream [ ≤ = 300 bp]; red, distal intergenic [> 500 bp from TSS and > 300 bp from TES]. (**H**) Heatmap of RNAPII signals in regions ± 3 kb from the R-loop on the genomes of yeast cells. The RNAPII data are from RNAPII ChIP sequencing from Morselli et al. [64]

To further study the potential function of R-loops during meiosis, we then detected genome-wide R-loops by our recently developed method ssDRIP-seq (single-strand DNA ligation-based library construction from DNA:RNA hybrid immunoprecipitation, followed by sequencing; see methods) [30–33]. ssDRIP-seq analysis of wild-type budding yeast cells in the vegetative growth stage (Fig. 2A-C) identified a total of 952 R-loop peaks (867 peaks for Watson R-loops [wR-loops] and 919 peaks for Crick R-loops [cR-loops]), collectively covering approximately 3.7% of the yeast genome (Fig. 2D). Most of the R-loops ranged between 250 and 700 base pairs (bp) in length (Fig. 2E) and were highly enriched with GA bases (Fig. 2F). RNase H treatment before DNA:RNA hybrid immunoprecipitation abolished the ssDRIP signal, which further supports the specificity of ssDRIP-seq (Fig. 2C).

We next assessed the genomic distribution of the R-loops and found that more than 60% of the R-loops resided in promoter-proximal regions (± 0.5 Kb from the transcription start site [TSS]) (Fig. 2G), which is consistent with a previous report [34]. The observation that the strongest signals for R-loops were coincident with RNA polymerase II (RNAPII) occupancy (Fig. 2H) is similar to previous reports, in which R-loops were found to be associated with gene transcription [34, 35]. T-based enrichment in Fig. 2F may also be associated with some promoter motifs, such as TATA boxes [36]. In addition to promoter regions, R-loops at the transcription end site (TES) were enriched (Additional file 1: Fig S1A), again similar to previous results [34]. Furthermore, the localization of R-loops on the DNA strand is strongly associated with the orientation of gene transcription, as wR-loops were enriched in genes on the DNA Watson strand; in contrast, cR-loops were enriched on Crick DNA strand genes (Additional file 1: Fig S1B, C). In addition to these enriched regions, we also detected R-loops at diverse genome regions, including telomeres, Ty elements, and loci for tRNA, rRNA, and small nucleolar RNA (Additional file 1: Fig. S2), which is consistent with previous reports [34, 37, 38]. Thus, ssDRIP-seq can efficiently detect R-loops that occur throughout the genome and reflect strand-specific information in budding yeast.

### R-loop profile during yeast sporulation

**Fig. 3:**
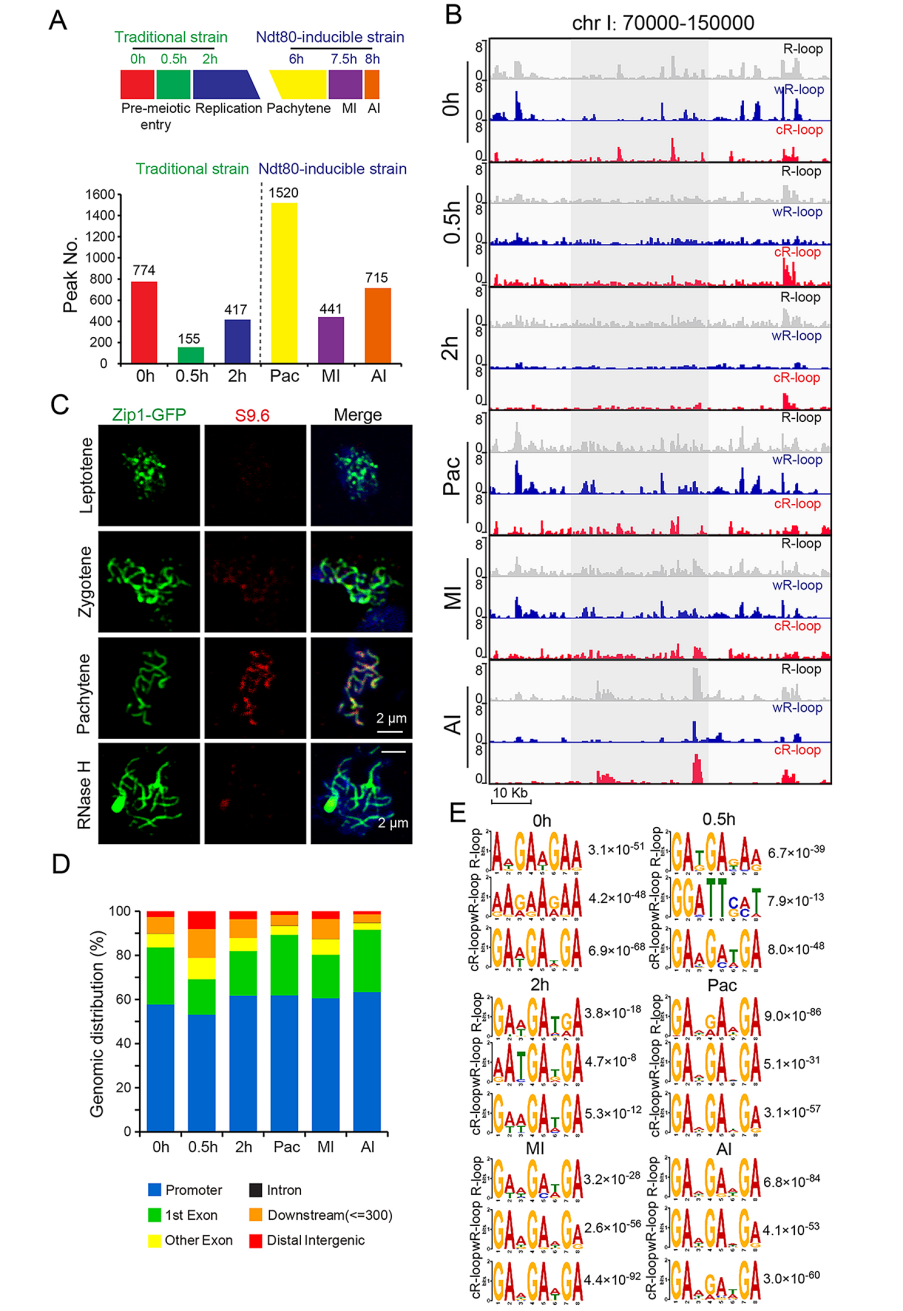
Genome-wide detection of R-loops during meiosis by ssDRIP-seq. (**A**) The number of overlapping ssDRIP-seq peaks from two biological repeats during yeast meiosis (0 h, 0.5 h, and 2 h in traditional synchronized SK1 strains [41]; 6 h, pachytene stage, Pac; 7.5 h, metaphase I, MI; and 8 h, anaphase I, AI in *NDT80*-induced synchronized SK1 strains [39]). The ssDRIP-seq peaks were analysed by peak calling strategies of MACS2 with default settings to call narrow R-loop peaks [63]. (**B**) Snapshot of the ssDRIP-seq data during yeast sporulation (0 h, 0.5 h, 2 h, Pac, MI, AI), including R-loop (grey), wR-loop (blue) and cR-loop (red) in a representative genomic region (chrI:70,000–150,000). (**C**) R-loop loads onto meiotic chromosomes during prophase I. Yeast cells were incubated in SPM and harvested at 6 h, and meiotic chromosomes were spread for immunofluorescence (Zip1-GFP, green; S9.6, red). Nuclei were stained with DAPI (blue). RNase H treatment served as a negative control. (**D**) The genomic distribution of R-loops during meiosis (0 h, 0.5 h, 2 h, Pac, MI, AI) that were identified by ChIPseeker. Various genomic regions are colour coded according to the labels at the bottom (blue, promoter; green, 1st exon; yellow, other exon; black, intron; orange, downstream [≤ 300 bp]; red, distal intergenic [> 500 bp from TSS and > 300 bp from TES]). (**E**) DNA motif in the peak regions of unstranded R-loops, wR-loops and cR-loops during meiosis (0 h, 0.5 h, 2 h, Pac, MI, AI) that were identified by MEME-ChIP. E-values are provided on the right

Having established the capacity of ssDRIP-seq to accurately identify multiple R-loop subtypes across the yeast genome, we next applied our method to characterize the SK1-background budding yeast strain [39, 40] at an extensive series of meiotic stages to dissect the functional impacts of R-loops in meiosis (Fig. 3 and Additional file 1: Fig. S3). Two separate meiosis experiments were performed according to a previous report [41] (Fig. 3A): the first used traditional synchronization procedures and focused on early meiotic stages (0 h, 0.5 h, and 2 h) [41], and the second time course used an oestrogen-activatable variant of the *NDT80* transcription factor [39], which allowed synchronous progression through meiosis (6 h, pachytene stage, Pac; 7.5 h, metaphase I, MI; 8 h, anaphase I, AI). Thus, our ssDRIP-seq data for a course of meiosis development allowed us to monitor the dynamic formation, maintenance, and resolution of R-loops at specific genomic regions as cells progressed through meiosis (Fig. 3B). Generally, the R-loop peak size detected for meiotic cells was similar to the peaks initially characterized in the vegetative stage (Fig. 2A-C, and Additional file 1: Fig. S3C). There were obvious dynamic changes in R-loops throughout the meiosis development course (Fig. 3A, and Additional file 1: Fig. S4). In addition, we found that R-loop signals stained by the S9.6 antibody, which is effective in recognizing DNA:RNA hybridization in R-loops [42], were first detected at the zygotene with foci signal, then highly expressed at the pachytene stage and co-localized with the synapsed chromosome axes during meiosis in wild-type yeast cells (Fig. 3C), which further supports the ssDRIP-seq result.

Our data illustrating the dynamic landscapes of R-loops in cells at diverse meiotic stages support the plausibility that R-loops may functionally regulate meiosis. The dynamic genomic distributions of R-loops throughout meiosis were analysed, revealing some general trends common to almost all phases of meiosis. For example, most R-loops reside in promoter regions and gene body regions, which was consistent with our initial analysis of vegetative stage cells (Fig. 3D), and we detected that the GA bases in R-loop regions were enriched (Fig. 3F). These common features again support potential transcription-level regulatory impacts for R-loops in meiotic genomes. To further confirm this hypothesis, we analysed the relationship between R-loops and gene transcription using published RNA-seq data [41] (Additional file 1: Fig. S4A-C). We found that R-loops at 0 h, 2 h, and Pac mainly accumulated in the promoter and gene body regions of highly expressed genes (top 5%) at their corresponding time points (Additional file 1: Fig. S4A-C). Furthermore, we compared the R-loops from the vegetative growth stage (YPD) and premeiotic entry stage (0 h) by using DESeq2 algorithms and found that the upregulated R-loops at 0 h were highly associated with meiosis-related genes and that the downregulated R-loops at 0 h were correlated with vegetative growth genes (Additional file 1: Fig. S4D, E). Thus, R-loops are highly associated with gene transcription during meiosis.

We also detected several obvious meiosis-stage-specific trends for R-loop distribution (Fig. 3D, E). Genome distribution analysis revealed that meiotic cells at 0.5 h after transfer to sporulation medium contained dramatically more R-loops in distal intergenic regions (> 500 bp from TSS and > 300 bp from TES) compared to that of other meiotic stages (Fig. 3D, Additional file 1: Fig. S3D). Moreover, we detected T base enrichment in the R-loops at these two time points (Fig. 3E). These results suggested that, except for transcriptional regulation, R-loops may play some special roles during the early stage of yeast meiosis.

### Some R-loops are associated with meiotic DSBs during yeast meiosis

**Fig. 4:**
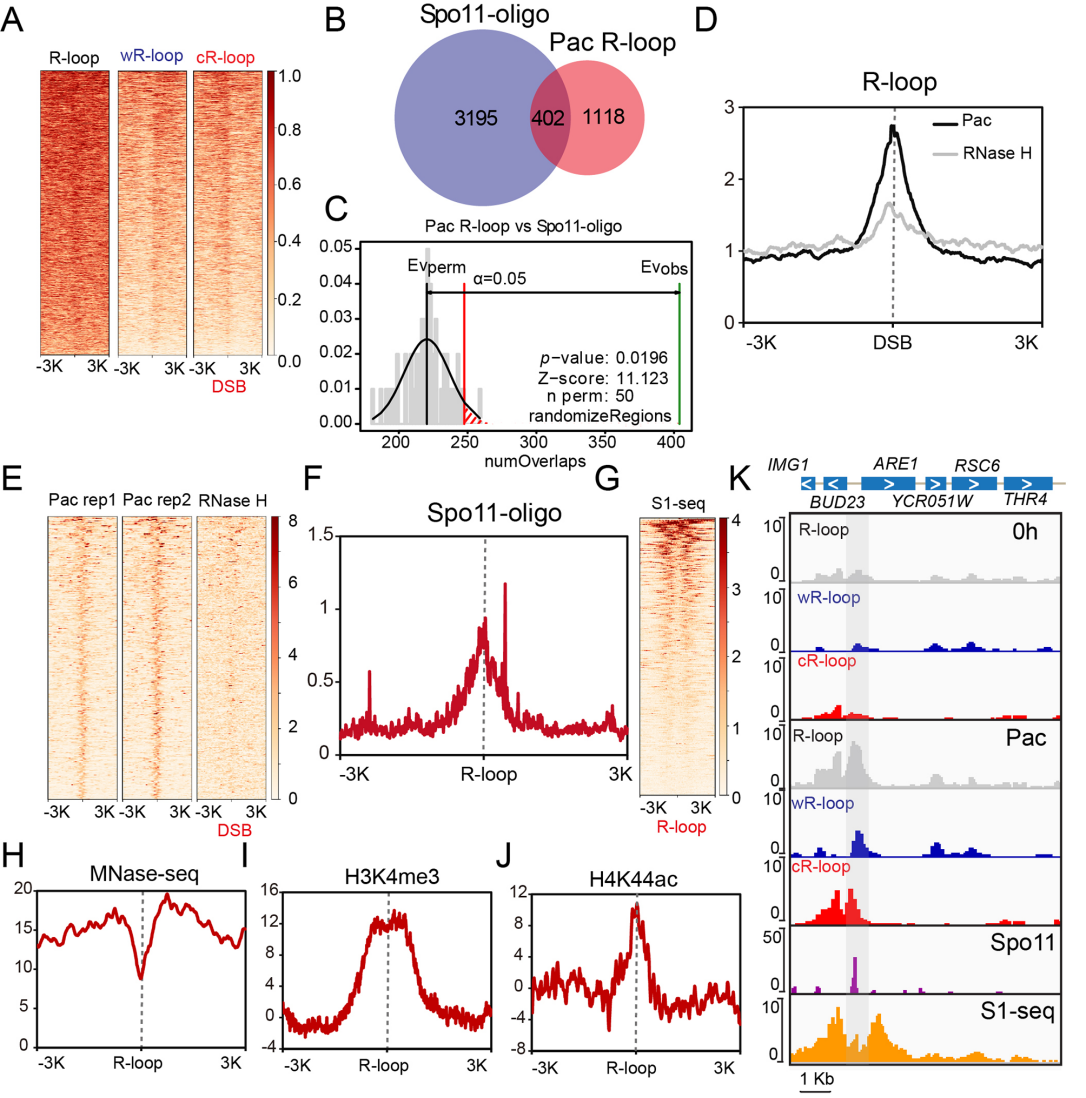
R-loops co-localize with meiotic DSB hotspots in pachytene cells. (**A**) Heatmap of ssDRIP-seq signals (R-loop, wR-loop and cR-loop) in regions ± 3 kb from previously confirmed meiotic DSB distribution on the genomes of pachytene cells from Spo11-oligo sequencing from Pan et al. data during yeast meiosis [7]. (**B**) Venn plots showing the overlap of Spo11-oligo-seq peaks [7] and R-loop peaks at the pachytene stage. Pac, pachytene. (**C**) Permutation test of colocalization between Spo11-oligo-seq peaks and R-loop peaks at the pachytene stage. Pac, pachytene; Evobs, evaluation observe; Evperm, evaluation permutation. (**D**) R-loop profile relative to some meiotic DSB sites. The signal intensity profiles of ssDRIP-seq at the pachytene stage and RNase H-treated ssDRIP-seq in regions ± 3 kb from the centre of the colocalized Spo11-oligo-seq signal region in (**B**). (**E**) Pachytene stage R-loops are enriched in some meiotic DSB sites. Heatmap of the ssDRIP-seq signals at the pachytene stage and RNase H-treated ssDRIP-seq signals at regions ± 3 kb from the centre of the colocalized Spo11-oligo-seq signal region in (**B**). (**F**) The signal intensity profiles of Spo11-oligo-seq in regions ± 3 kb from the centre of meiotic DSB overlapping R-loops detected in our ssDRIP-seq analysis at the pachytene stage. (**G**) DNA:RNA hybrids could form at ssDNA ends of meiotic DSBs. Heatmap of S1-seq signals [9] in regions ± 3 kb from meiotic DSB overlapping R-loops. (**H**) The signal intensity profiles of MNase-seq [65] in regions ± 3 kb from the centre of meiotic DSB overlapping R-loops detected in our ssDRIP-seq at the pachytene stage. (**I**) The signal intensity profiles of H3K4me3 [65] in regions ± 3 kb from the centre of meiotic DSB overlapping R-loops detected in our ssDRIP-seq analysis at the pachytene stage. (**J**) The signal intensity profiles of H4K44ac [65] in regions ± 3 kb from the centre of meiotic DSB overlapping R-loops detected in our ssDRIP-seq analysis at the pachytene stage. (**K**) A representative genomic region showing R-loop signals sharing overlap with meiotic DSBs detected in Spo11-oligo-seq and S1-seq analysis. The wR-loop and cR-loop asymmetrically localized one side of the hotspot centres at the pachytene stage.

It has been reported that DNA:RNA hybrids can form at ssDNA ends of meiotic DSBs to regulate meiotic recombination [26]. We examined the relationship between R-loops and DSB sites derived from high-resolution genome-wide DSB mapping based on Spo11-oligo sequencing [7]. We found that R-loops showed strong enrichment at hotspot centres at the pachytene stage, and the wR-loop and cR-loop showed a slight tendency to localize one side of hotspot centres at the pachytene stage (Fig. 4A, K). These results indicate that R-loops might form at meiotic DSB regions, which is consistent with a previous report [26].

Because R-loops have multiple roles and can be produced in multiple ways [16, 17, 21, 43], we wanted to know which kind of R-loop is associated with those DSB hotspots. A permutation test indicated that R-loop peaks at the pachytene stage showed colocalization relationships with Spo11-oligo peaks, and 26.45% (402/1520) R-loop peaks co-localized with meiotic DSB hotspots, which were termed meiotic DSB overlapping R-loops (Fig. 4B, C). R-loops at the pachytene stage were strongly enriched at these associated hotspot centres (402/3597, 11.17%) (Fig. 4D, E), and wR-loops and cR-loops also showed a slight tendency to localize one side of these associated hotspot centres (402/3597) (Additional file 1: Fig. S5). The Spo11-oligo-seq signals were co-localized with the meiotic DSB overlapping R-loop regions (Fig. 4F, K). Furthermore, the S1-seq signal, which marks the DSB resection endpoints by removing the ssDNA tails of resected DSBs, preparing the ends for ligation with the 5’ biotinylated adaptor and deep sequencing [9], was distributed outside the signals of these meiotic DSB overlapping R-loops (Fig. 4G, K), further supporting that DNA:RNA hybrids could form at the ssDNA ends of meiotic DSBs. As some meiotic recombination hotspots exhibit an increase in micrococcal nuclease (MNase) accessibility [7, 44], we further detected nucleosome occupancy at meiotic DSB overlapping R-loop regions and found that these DSB hotspot-associated R-loops showed strong enrichment at nucleosome-depleted regions (Fig. 4H). In support of these results, we found that meiotic DSB overlapping R-loops were also correlated with H3K4me3 and H4K44ac (Fig. 4I, J), which facilitate meiotic recombination and are enriched at DSB hotspots [45–47]. Therefore, these results suggest that R-loops might be associated with some meiotic recombination during yeast meiosis.

### DSB hotspot-associated R-loops are newly generated

**Fig. 5:**
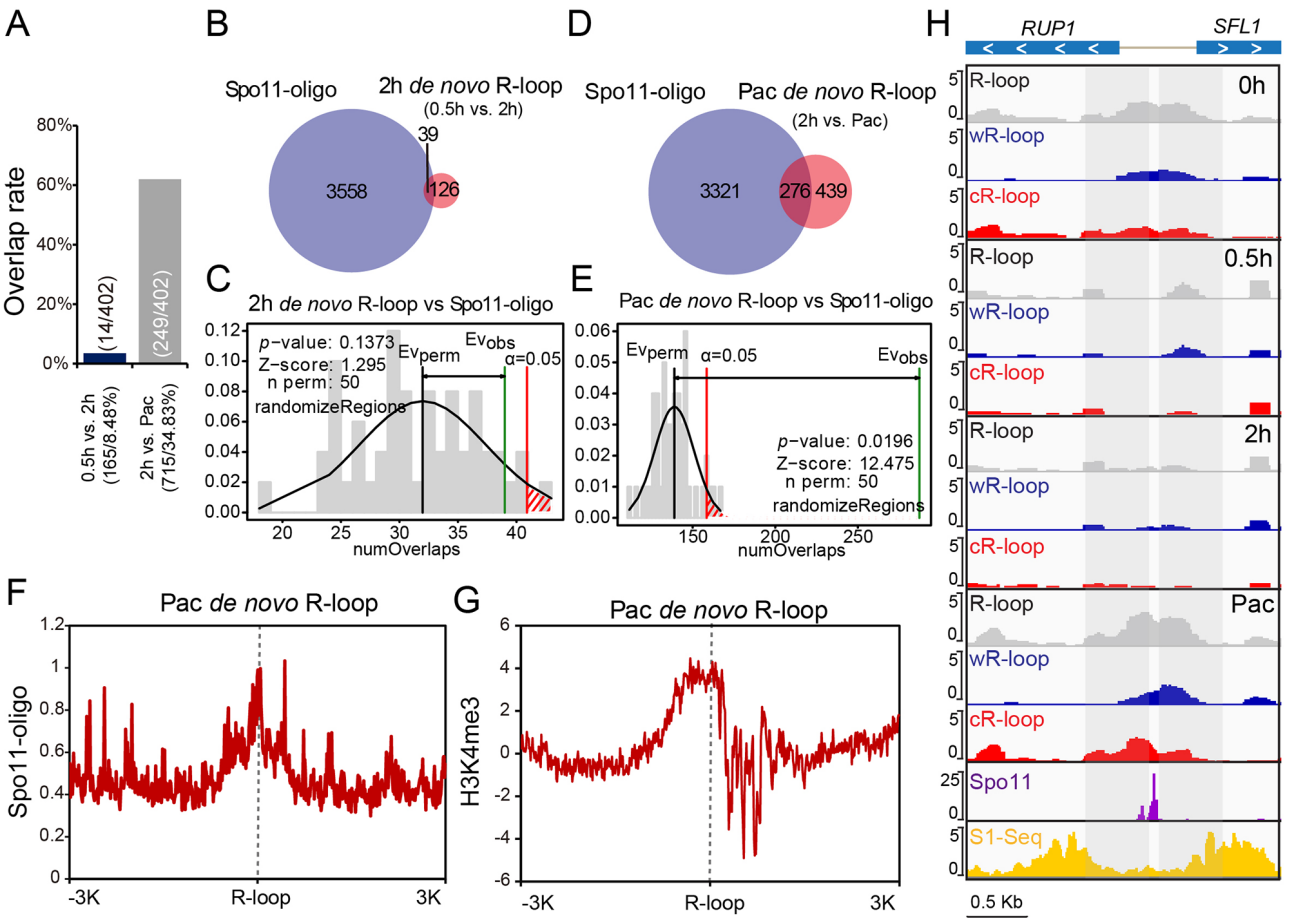
DSB hotspot-associated R-loops are newly generated at the early stage of meiosis (**A**) Plot shows the overlap rate of meiotic DSB overlapping R-loop peaks and *de novo* R-loop peaks at the early stage of yeast meiosis (2 h *de novo* R-loop: 0.5 h vs. 2 h; Pac *de novo* R-loop: 2 h vs. Pac). The number of 2 h *de novo* R-loop peaks was 165, and 14 peaks (8.48%) overlapped with meiotic DSB overlapping R-loops. The number of Pac *de novo* R-loop peaks was 715, and 249 peaks (34.83%) overlapped with meiotic DSB overlapping R-loops. Pac, pachytene. (**B**) Venn plots showing the overlays of Spo11-oligo-seq peaks [7] and 2 h *de novo* R-loop peaks (0.5 h vs. 2 h) of yeast meiosis. (**C**) Permutation test of colocalization between Spo11-oligo-seq peaks and 2 h *de novo* R-loop peaks. Evobs, evaluation observe; Evperm, evaluation permutation. (**D**) Venn plots showing the overlays of Spo11-oligo-seq peaks [7] and Pac *de novo* R-loop peaks (2 h vs. Pac) of yeast meiosis. Pac, pachytene. (**E**) Permutation test of colocalization between Spo11-oligo-seq peaks and Pac *de novo* R-loop peaks.  Pac, pachytene; Evobs, evaluation observe; Evperm, evaluation permutation. (**F**) The signal intensity profiles of Spo11-oligo-seq [7] in regions ± 3 kb from the centre of Pac *de novo*  R-loops (2 h vs. Pac) detected in our ssDRIP-seq analysis. Pac, pachytene. (**G**) The signal intensity profiles of H3K4me3 [65] in regions ± 3 kb from the centre of Pac *de novo* R-loops (2 h vs. Pac) detected in our ssDRIP-seq analysis. Pac, pachytene. (**H**) A representative genomic region showing *de novo* R-loop signals (2 h vs. Pac) at the pachytene stage sharing overlap with meiotic DSBs detected in Spo11-oligo-seq and S1-seq analysis. The *de novo* wR-loop and cR-loop at the pachytene stage localized one side of the hotspot centres at the pachytene stage. Pac, pachytene.

As R-loops were dynamically changed at the early stage of meiosis and exhibited a peak at the pachytene stage (Fig. 3, Additional file 1: Fig. S6), we wondered whether these meiotic DSB overlapping R-loops were newly generated. To test this hypothesis, we compared the R-loop peaks at the early stage of meiosis (0.5 h vs. 2 h, termed 2 h *de novo* R-loops; 2 h vs. Pac, termed Pac *de novo* R-loops) by using DiffBind algorithms based on the reproducible peaks from two biological repeats and obtained the 2 h and Pac *de novo* R-loop peaks (Additional file 1: Fig. S6). Then, we compared the meiotic DSB overlapping R-loops with these *de novo* R-loop peaks (Fig. 5A) and found that most of these R-loops were newly generated at the pachytene stage of meiosis (2 h vs. Pac, but not 0.5 h vs. 2 h) (Fig. 5A). In addition, Pac *de novo* R-loop peaks (2 h vs. Pac) were also co-localized with Spo11-oligo-seq peaks (Fig. 5D, E), while the overlap rate between 2 h *de novo* R-loops (0.5 h vs. 2 h) and Spo11-oligo-seq peaks was low (Fig. 5B, C). Furthermore, the Spo11-oligo-seq and H3K4me3 signals showed enrichment at centres of Pac *de novo* R-loops (Fig. 5F, G). Therefore, DSB hotspot-associated R-loops are newly generated.

Given that many meiotic DSB hotspots occur in nucleosome-depleted gene promoters [7] and that R-loops are highly associated with gene transcription during meiosis (Fig. 3D, Additional file 1: Fig. S4D, E), the co-localization between R-loops and meiotic DSB hotspots at the early stage of meiosis may be due to a spatial overlap between R-loop-associated high gene transcription and nucleosome-depleted gene promoters. To examine this, we first identified pachytene-upregulated genes using published RNA-seq data [41] and detected the relationship between pachytene-upregulated gene promoter regions and meiotic DSB overlapping R-loops. Only 16.4% (66/402) of meiotic DSB overlapping R-loops co-localized with pachytene-upregulated gene promoter regions (Additional file 1: Fig. S7A). Next, we examined the relationship between Pac *de novo* R-loops and the pachytene stage upregulated genes (Pac vs. 2 h) [41] and found that only 93 of 715 pachytene *de novo* R-loops co-localized with the pachytene upregulated gene (Pac vs. 2 h) promoter and gene body regions (Additional file 1: Fig. S7B, C). Thus, the increased R-loop signal at the pachytene stage may not be associated with transcription levels during meiosis, and the correlation between the meiotic DSB hotspots and R-loops may not originate from highly expressed genes.

To further study the relationship between R-loops and meiotic recombination, we eliminated meiotic DSBs in *rnh1/rnh201Δ* cells by depleting *SPO11* [3, 4] and found that *rnh1/rnh201/spo11Δ* displayed similar meiotic procession to that of the WT cells (Additional file 1: Fig. S8A). R-loop accumulation may disrupt meiosis by impairing meiotic recombination because loss of Spo11 function can rescue the meiotic progress delay of *rnh1/rnh201Δ* cells, similar to a previous report [26]. In addition, some R-loop signals near meiotic DSB sites showed decreased in *SPO11* knockout cells (Additional file 1: Fig. S8B), while the knockout of *SPO11* has little effect on some stronger R-loop regions out of the meiotic DSB overlapping R-loops (Additional file 1: Fig. S8C). Thus, some R-loops may be associated with Spo11 function during yeast meiosis.

### Transcription-replication conflict-induced R-loops correlated with meiotic DSBs

**Fig. 6:**
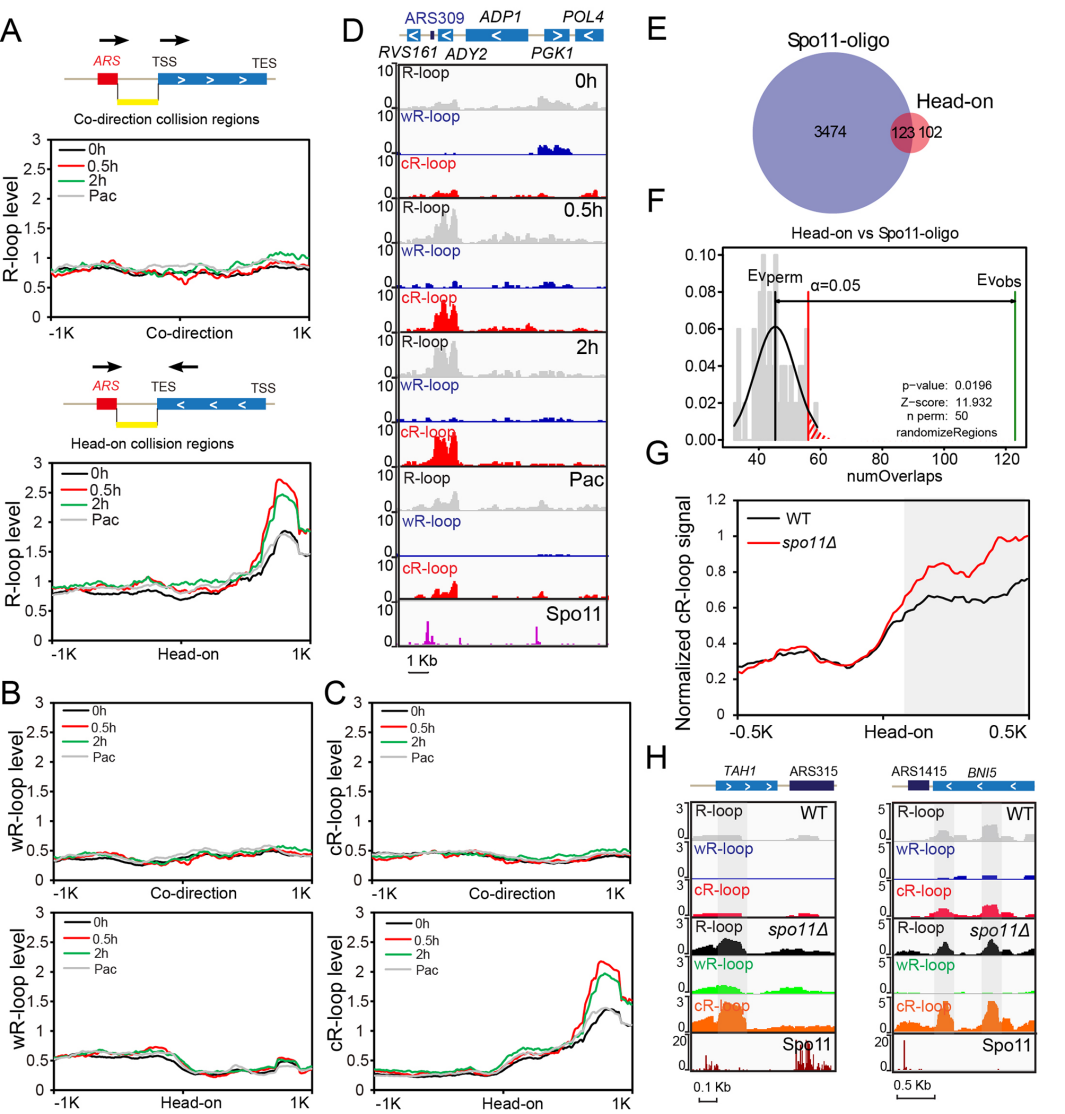
Transcription-replication conflict-induced R-loops correlated with meiotic DSB formation. (**A**) The signal intensity profiles of R-loop peaks (0 h, 0.5 h, 2 h, Pac) in regions ± 1 kb from the centre of codirectional collisions (the orientation of gene transcription and DNA replication in the same direction) and head-on collisions (the orientation of gene transcription and DNA replication in the opposite direction) identified with the orientation of gene transcription and the localization of ARS regions, respectively. The annotations of the codirectional collision regions and head-on collision regions are shown in (**A**). (**B**) Metaplots of wR-loop peaks centred on codirectional collisions and head-on collisions identified with the orientation of gene transcription and the localization of ARS regions. (**C**) Metaplots of cR-loop peaks centred on codirectional collisions and head-on collisions identified with the orientation of gene transcription and the localization of ARS regions. (**D**) A representative genomic region covering the *ARS309* and *ADY2* head-on collision region (chrIII:130,736 − 133,314), showing R-loop signals (0 h, 0.5 h, 2 h, Pac) sharing overlap with meiotic DSBs [7]. **(E)** Venn plots showing the overlays of Spo11-oligo-seq peaks [7] and head-on collision regions. **(F)** Permutation test of colocalization between Spo11-oligo-seq peaks and head-on collision regions. Evobs, evaluation observe; Evperm, evaluation permutation. (**G**) The signal intensity profiles of cR-loop peaks in regions ± 0.5 kb from the centre of head-on collisions in the WT and *spo11Δ* strains at 2 h after sporulation. cR-loop signal intensities were calculated and normalized by subtracting basal levels of signal intensities. (**H**) A representative genomic region covering the *ARS3315* and *TAH1* head-on collision region,* ARS1415* and *BNI5* head-on collision region in the WT and *spo11Δ* strains at 2 h after sporulation, showing R-loop signals sharing overlap with meiotic DSBs.

It has been reported that conflicts between transcription and replication promote R-loop formation, and these conflicts are potent sources of DNA damage [48]. Furthermore, premeiotic DNA replication is known to be mechanistically coupled to the initiation of meiotic recombination [11, 12, 24]. We therefore speculated that transcription and meiotic DNA replication conflicts may somehow promote R-loop formation, potentially thereby contributing to the induction of meiotic DSBs. As the progression of replication forks and gene transcripts are dynamically regulated during meiosis, it is difficult to accurately measure the regions of transcription-replication conflicts. We annotated codirectional collision regions (the orientation of gene transcription and DNA replication in the same direction) and head-on collision regions (the orientation of gene transcription and DNA replication in the opposite direction) at a genome-wide scale based on the orientation of gene transcription and the localization of autonomously replicating sequence (ARS) regions (Fig. 6A). Then, we analysed the distribution of R-loops in transcription-replication codirectional collision regions and head-on collision regions during meiosis (Fig. 6A-C). Most obviously, we detected strong enrichment for R-loops positioned close to head-on collision regions (within 1 kb) at 0.5 and 2 h after transfer to sporulation medium; no enrichment of R-loops was detected near codirectional collision regions (Fig. 6A-D). Furthermore, R-loop signals near head-on collision regions were decreased at the pachytene stage (Fig. 6A-D). As ssDRIP-seq can reflect strand-specific information of R-loops, we further analysed the strand specificity of the enriched R-loops close to head-on collision regions and found that most of the R-loops were localized on the Crick strand (Fig. 6B, C). At minimum, these results support that transcription-replication head-on collisions may promote R-loop formation during meiotic DNA replication.

Given that persistent R-loops promote genome instability [17, 21], we next examined the relationship between meiotic DSBs and R-loops induced by head-on collisions. Spo11 is a key transesterase-like enzyme that induces meiotic DSBs [3, 4]. First, we found that Spo11-oligo signals could be broadly observed near transcription-replication head-on collision-induced R-loop regions (Fig. 6D, H), and approximately 123 of 255 transcription-replication head-on conflicts were close to Spo11 cut sites (Fig. 6E, F). Then, we deleted *SPO11* and detected the R-loop signal in the *spo11Δ* strain at 2 h after sporulation (Fig. 6G, H, Additional file 1: Fig. S9) and found that the *spo11Δ* strain accumulated a higher signal of cR-loops near head-on collision regions than that of the WT strain (Fig. 6G, H); this result supports the idea that the Spo11 protein may function to somehow resolve the R-loops caused by head-on collision during meiosis.

As previous reports showed that Rnh1 and Rnh201 function to specifically remove RNA from DNA:RNA hybrids to eliminate R-loops [27–29], we further compared R-loops in *spo11Δ* cells and *rnh1*/*rnh201Δ* cells at the early stage of meiosis (0.5 and 2 h) (Additional file 1: Fig. S9B-E). We found that at the early stage of meiosis, the distribution of R-loops in the *rnh1*/*rnh201Δ* strain was similar to that in the *spo11Δ* strain (Additional file 1: Fig. S9C, D), and principal component analysis (PCA) revealed that R-loops in the *rnh1*/*rnh201Δ* strain were clustered with those in the *spo11Δ* strain (2 h) (Additional file 1: Fig. S9E). These findings support that meiotic DSBs may function to somehow resolve the R-loops caused by head-on collision during meiotic DNA replication, and transcription-replication conflict-induced R-loops may be a source of meiotic DSBs.

### R-loops induced by transcription-replication conflicts are associated with Spo11

**Fig. 7:**
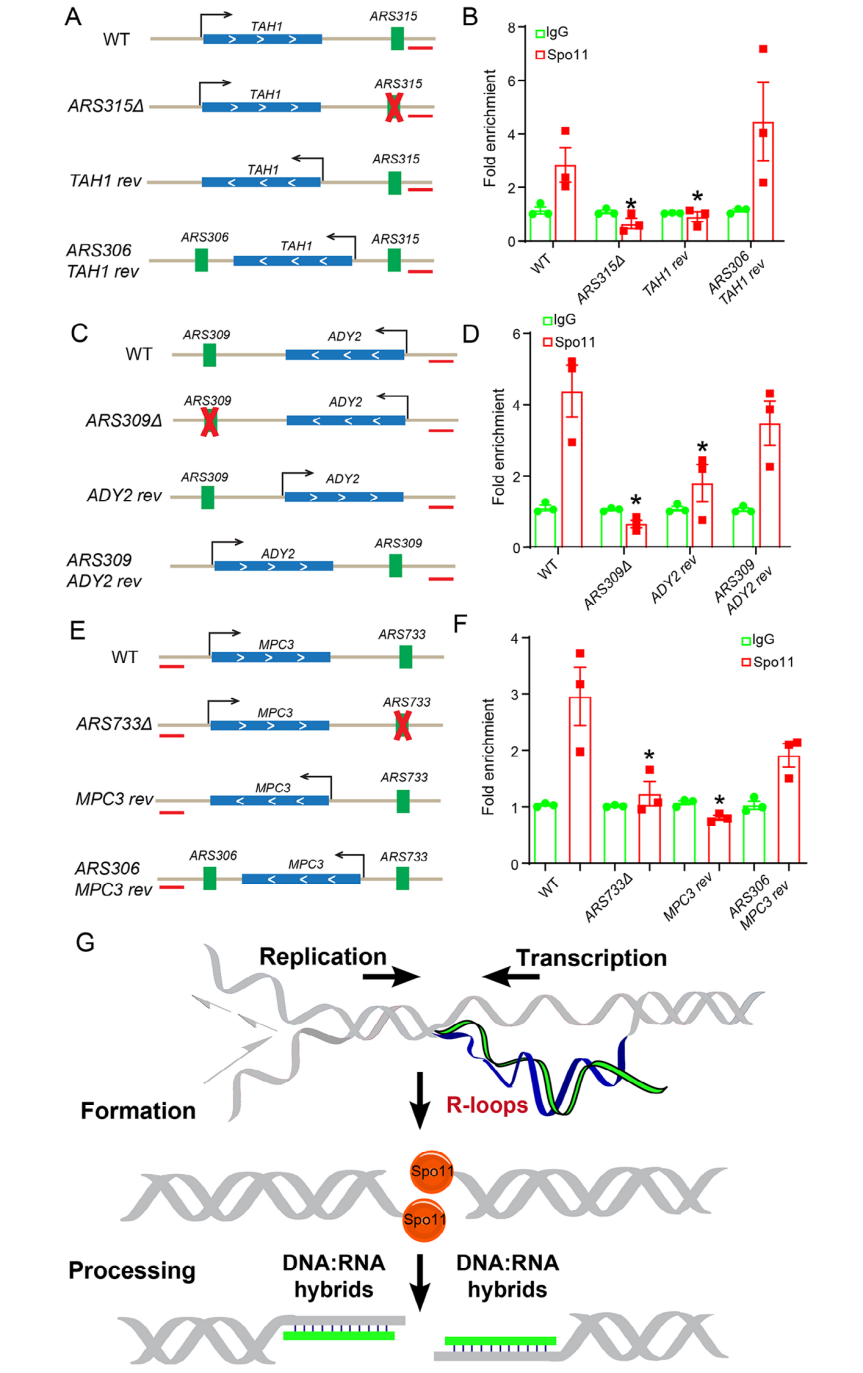
R-loops induced by transcription-replication conflicts determine some meiotic DSBs. (**A**) Schematic representation of the *ARS315*-*TAH1* head-on collision. *ARS315Δ* refers to a strain from which the *ARS315* sequence has been deleted; *TAH1 rev* refers to a strain in which the orientation of the *TAH1* locus was inverted; *ARS306-TAH1 rev* refers to a strain constructed by inserting the *ARS306* sequence into the *TAH1 rev* strain background to induce a new head-on collision between *ARS309 and TAH1*. The red bars indicate the Spo11 ChIP‒qPCR detected regions. (**B**) Spo11 ChIP‒qPCR of the WT, *ARS315Δ*, *TAH1 rev*, and *ARS306-TAH1 rev* strains at 2 h after sporulation. (**C**) Schematic representation of the ARS309-*ADY2* head-on collision. *ARS309Δ* indicates the strain deleted *ARS309* region, *ADY2 rev* indicates the strain changed the *ADY2* orientation and *ARS309-ADY2 rev* indicates the strain changed the *ARS309-ADY2* orientation. (**D**) Spo11 ChIP‒qPCR in the WT, *ARS309Δ*, *ADY2* reverse and *ARS309-ADY2* reverse strains at 2 h after sporulation. (**E**) Schematic representation of ARS733-*MPC3* head-on collision. *ARS733Δ* indicates that the strain deleted the *ARS733* region, *MPC3 rev* indicates that the strain changed the *MPC3* orientation, and *ARS306-MPC3 rev* indicates that the *MPC3 rev* strain inserted the ARS306 sequence to create a new head-on collision. (**F**) Spo11 ChIP‒qPCR in the WT, *ARS733Δ*, *MPC3* reverse and *ARS733-MPC3* reverse strains at 2 h after sporulation. (**G**) Dual roles of R-loops in meiotic DSB formation and processing

To examine any functional role(s) for transcription-replication head-on collision in DSB formation, we next asked whether the disruption of head-on collision is sufficient to eliminate meiotic DSB formation. By checking the distribution of Spo11-oligo sequencing data near transcription-replication head-on collision regions, we selected five R-loop accumulated head-on collision regions in the *SPO11-9*$\times$*myc* strain and deleted their respective ARS sequences or inverted the orientation of a gene transcription template locus; collectively, these genetic manipulations should resolve the particular transcription-replication head-on collisions we focused on. Because the DSB site in these regions was not hot enough to be detected by southern blotting and Spo11-oligo-seq, we monitored for any Spo11 signal near the head-on collision of interest in various deletion strains by Spo11 ChIP‒qPCR at 2 h after sporulation. First, note that genetic manipulation of these strains did not affect their sporulation process. ChIP‒qPCR analysis revealed significantly decreased Spo11 binding in the tested regions of several ARS deletion and gene reverse strains at 2 h after sporulation (Fig. 7A-F). These findings show that abolishment of transcription-replication head-on collisions may prevent Spo11 binding to chromatin.

Extending this, we next generated strains in which new head-on collisions were induced. Specifically, we introduced an ARS into a strain with an inverted *TAH1* locus (Fig. 7A and B); we also generated a strain with an inverted locus comprising an ARS and the *ADY2/MPC3* locus (Fig. 7C-F). Moreover, ChIP‒qPCR analysis revealed that a Spo11 signal was present at these induced head-on collision regions 2 h after sporulation (Fig. 7A-F). These experiments illustrate that experimentally altering chromatin structures enables successful elimination or reconstitution of particular Spo11 binding. Furthermore, our results show that at least some meiotic DSBs may originate from R-loops, which are associated with transcription-replication head-on collisions during meiosis.

## Discussion

The distribution of meiotic DSBs across the genome is not random and occurs at relatively high frequencies in some genomic regions known as hotspots [10–12, 49]. Many factors have been reported to be correlated with these meiotic hotspots, including histone modifications, TFs, the meiotic chromatid cohesin complex, and chromatin structure [7, 10, 11, 13–15]. However, none of these studies uncovered any conserved consensus sequence for these hotspots [7, 11, 14], suggesting that the hotspots are not simply determined by DNA sequence. To identify the hotspots, the targeting of Spo11 or some other recombination initiation proteins to a specific site is sufficient to stimulate meiotic recombination [50–52]; in addition, tethering Spp1, a component of the H3K4 methylase COMPASS complex, to recombinationally cold regions could induce meiotic DSB formation [45]. However, the mechanisms underlying meiotic recombination hotspot determination are still largely unknown.

Premeiotic DNA replication is mechanistically coupled to the initiation of meiotic recombination [11, 12, 24] because blocking premeiotic DNA replication prevents meiotic DSB formation in a replication-checkpoint–independent manner [24]. The features of chromatin structure also play a role in determining meiotic recombination event initiation, and the vast majority of breaks occur at or near many potential transcription promoters [13]. Elevated R-loop levels have been reported to be associated with increased DNA damage and genome instability from yeast to human cells [16, 21, 43]. Our results showed that head-on collisions between transcription and meiotic DNA replication could induce R-loop formation during meiotic DNA replication (Fig. 6). Higher T base enrichment in the 0.5 and 2 h R-loops may be caused by the enrichment of R-loops near the head-on collision regions, as some ARS regions contained a general 5′-(T/A) TTTAT (A/G) TTT (T/A)-3′ motif [53]. As deleted *SPO11* caused a slight accumulation of cR-loop signals near head-on collision regions (Fig. 6C, D, Additional file 1: Fig. S10A), some meiotic DSB hotspots may originate from those R-loops that are associated with transcription-replication head-on collisions during meiosis (Fig. 6).

To examine the effect of transcription-replication head-on collisions on meiotic DSB formation, we generated strains to abolish or induce transcription-replication head-on collisions and found that altering chromatin structures enables successful elimination or reconstitution of some meiotic DSB hotspots (Fig. 7A-F). As deleting origins of replication causes a delay in assembly of the DSB machinery [25] and changing the orientation of transcription impacts the loop-axis organization [54] that further impairs the distribution of DSB proteins [55], we could not absolutely exclude indirect effects of altering chromatin structures on meiotic DSB machinery assembly.

Given that Spo11 ChIP‒qPCR could only reflect Spo11 binding and that Spo11 mainly cuts DNA after entering meiosis [56, 57], we speculate that two steps occurred for conflict loop resolution during meiosis. First, transcription-replication conflict may be associated with Spo11 binding during meiotic DNA replication to establish the environment for Spo11 activity later [25], and then the preloaded Spo11 may facilitate transcription-replication conflict resolution after entering meiosis. Indeed, a small portion of the genome shows delayed replication at the zygotene stage [58], which may be associated with the transcription-replication conflict. We also noticed that meiotic DSBs were broadly distributed near the transcription-replication conflict (Figs. 6B and D, and 7A-F). Given that Spo11 triggers DNA breaks via its dimerization [59], it is unlikely that the Spo11 complex could directly cut at R-loop sites as both DNA strands are separate, and cleavage may randomly occur at adjacent DNA pieces in the R-loop regions.

Spo11-oligos sequencing led to the identification of more than 3,000 DSB hotspots in yeast, but only a small subset of meiotic DSBs overlap with R-loops. Thus, although we have uncovered a connection between transcription-replication collision and Spo11 binding to chromatin, many DSB hotspots apparently do not result from transcription-replication head-on collision, suggesting that some other factors may also be involved in the determination of meiotic DSB hotspots.

Aside from promoting DNA damage formation, R-loops have also been reported to be associated with meiotic DSB recombination [26]. During yeast sporulation, DNA:RNA hybrids have been reported to form at ssDNA ends of meiotic DSBs and regulate both crossover and noncrossover recombination by affecting homologue bias during meiosis [26]. We also found that some R-loops are associated with meiotic DSBs during meiosis and that the elimination of meiotic DSBs can rescue the meiotic progress delay of *rnh1/rnh201Δ* cells (Figs. 3 and 4 and Additional file 1: Fig. S8). However, there are some small discrepancies between our study and Yang’s work [26]. For example, the spore viability of the *rnh201Δ* strain in this work showed a slight decrease compared with that of the control groups (Fig. 1D), while the knockout of *RNH201* induced little effect on spore viability in Yang’s work [26]. They found that approximately 70% of DNA:RNA hybrids co-localized with RPA foci in the *rnh1/rnh201/hpr1Δ* strain during meiosis [26]; however, only approximately 25% of R-loops at the pachytene colocalized with Spo11-oligo peaks in this work (Fig. 4B). Moreover, we found that R-loop immunostaining signals stained by the S9.6 antibody could be detected at the zygotene with foci signal and co-localized with the synapsed chromosome axes at the pachytene in wild-type yeast cells (Fig. 3C). However, Yang et al. could not observe the R-loop signals in wild-type strains [26]. These discrepancies may be due to a difference in strains or methods. For example, we used a higher primary antibody concentration (1:50 in this work vs. 1:1000 in Yang’s work [26]) for R-loop immunostaining, and R-loops may be more stable in the *rnh1/rnh201/hpr1Δ* strain in Yang’s work [26] than in the wild-type strains used for ssDRIP-seq in our work (Fig. 3). However, both our and Yang’s data support that R-loops participate in meiotic DSB processing.

## Conclusions

In summary, we profiled genome-wide R-loops by using ssDRIP-seq during yeast meiosis and showed obvious dynamic changes in R-loops throughout meiosis. We found that multiple *de novo* R-loops at the pachytene stage were associated with meiotic DSB hotspots during meiosis. Transcription-replication head-on collisions during meiotic DNA replication may promote R-loop formation, and these R-loops may further promote meiotic DSB formation. Furthermore, some meiotic DSB hotspots can be eliminated by reversing the direction of either transcription or replication and reconstituted by reversing both of their directions. Therefore, in addition to meiotic DSB processing, R-loops may also participate in meiotic DSB formation.

## Methods

### Strains

All experiments were performed using diploid SK1 strains of budding yeast produced by mating appropriate haploids. Strains expressing C-terminal-tagged proteins, yeast deletion strains and gene reverse or ARS-gene reverse strains in Fig. 7 were constructed using a polymerase chain reaction (PCR)-based method. All yeast strains used in this study are described in Table S1.

### Sporulation conditions and meiotic nuclear division assays

Sporulation was induced using potassium acetate as previously described [60]. The strains were grown for 24 h in YPD medium (1% yeast extract, 2% peptone, and 2% glucose), diluted in liquid YPA medium (1% yeast extract, 2% peptone, and 2% potassium acetate) to OD600 = 0.3, and grown for 14 h for A14201-derived strains and 10 h for A14200-derived strains. Cells were washed 3 times, resuspended in sporulation medium (2% potassium acetate) to OD600 = 1.9 and sporulated at 30°C. For A14200-derived strains, sporulation samples were collected at 0 h, 0.5 h and 2 h. *GAL-NDT80 GAL4. ER* strains were released from pachytene arrest by the addition of 1 µM β-oestradiol (5 mM stock in ethanol; Sigma, E2758-1G) at 6 h. Meiotic nuclear divisions, representing sporulation efficiency, were visualized by staining chromosomal DNA with 1 µg/ml 4’,6-diamidino-2-phenylindole (DAPI); the samples were harvested at the indicated times and directly fixed in an equal volume of 100% ethanol for subsequent DAPI staining. Images were recorded and analysed under a Nikon Eclipse Ti microscope (Eclipse Ti-S; Nikon, Tokyo, Japan).

### ssDRIP-seq library construction

Nuclei were isolated from yeast cells by grinding well in liquid nitrogen, followed by SDS (final concentration: 0.5%)/proteinase K (final concentration: 0.1 mg/ml) treatment at 37°C overnight. A 1/4 volume of 5 M potassium acetate was added, and the tube was mixed and placed on ice for 10 ~ 20 min. Genomic DNA was extracted by the phenol‒chloroform method and precipitated with isopropanol. DNA fragmentation was performed at 37°C overnight using endonucleases (Alu I, DdeI, MboI, MesI and Rsa I; New England Biolabs, Ipswich, MA); the negative control was treated with RNase H (New England Biolabs) at 37°C overnight before endonuclease treatment. DRIP was performed as described previously [30]. The S9.6 antibody was purified from HB-8730 (ATCC, Manassas, VA). The DRIPed DNA samples were sonicated to a set size of 250 bp with an M220 Focused-ultra sonicator (Covaris, Woburn, MA, USA). The sonicated DNA was denatured at 95°C for 2 minutes and immediately placed on ice for 2 minutes to obtain ssDNA fragments. The first adapter (Adp1, GAT CGG AAG AGC ACA CGT CTG AAC TCC AGT CAC (i7) ATC TCG TAT GCC GTC TTC TGC TTG) was ligated to the 3’ end of the ssDNA using Adaptase (Swif Biosciences, Ann Arbor, MI, USA) via a highly efficient, proprietary reaction that simultaneously tails only the 3’ ends of ssDNA and ligates the first truncated adapter to the 3’ ends. This method avoids the bias inherent in random primer-based methods, as it ligates adapters in a sequence-independent manner. The extension step was performed using the primer paired to the first adapter, followed by a ligation reaction to add the second truncated adapter (Adp2, AAT GAT ACG GCG ACC ACC GAG ATC TAC AC (i5) ACA CTC TTT CCC TAC ACG ACG CTC TTC CG ATC T) to the 5’ ends. An indexing PCR step was performed to add the indexed sequence, and the library was amplified. The libraries were checked on an Agilent (Palo Alto, CA, USA) BioAnalyzer, followed by sequencing on an Illumina (San Diego, CA, USA) HiSeq X 10 system.

### ssDRIP-seq data analyses

For ssDRIP-seq, reads were aligned to the sacCer3 genome with Bowtie 2 using default settings, with all duplicates removed by Picard tools (http://broadinstitute.github.io/picard). The mapped reads (nonstranded R-loops) were divided into forwards reads (wR-loops, representing an R-loop formation containing ssDNA on the Watson strand and a DNA:RNA hybrid on the Crick strand) and reverse reads (cR-loops) by using samtools. Diffbind was used to analyse R-loop differences between samples. MACS2 was used to identify peaks with default parameters. For visualization, the aligned read files (Binary Alignment Map [BAM]) were converted to normalized coverage files (bigWig) using bamCoverage from deepTools (Ramírez et al., 2016). Normalization was performed using bamCoverage from deepTools, with read coverage normalized to 1× sequencing depth (also known as reads per genomic content) with renormalization by shuffled peaks (total R-loop peaks were shuffled randomly, and the 95% mean of the R-loop signal from each sample on shuffled peaks was used as the denominator) to eliminate disturbances from abnormally high-value regions. Snapshots of the data were constructed using the Integrative Genomics Viewer (IGV). Spearman’s rank correlation coefficients were calculated with plotCorrelation from deepTools using 500-bp bins. Heatmaps were generated with computeMatrix from deepTools. Metaplots were generated with deepTools.

### Chromatin immunoprecipitation (ChIP)

ChIP analysis of target proteins was performed using antibodies, largely as previously described [37]. Meiotic cells were cross-linked with formaldehyde (1%) for 25 min at room temperature. The pellets were resuspended in 400 µl of FA-1 lysis buffer [50 mM HEPES-KOH at pH 7.5, 140 mM NaCl, 1 mM EDTA at pH 8, 1% Triton X-100, 0.1% w/v sodium deoxycholate, plus CPI-EDTA 1 × (11,697,498,001, Protease inhibitor cocktail; Roche)], mixed with 500 µl of glass beads (G8772; Sigma, St. Louis, MO) and vortexed for 45 min at full speed at 4 °C. The glass beads were removed, and the cross-linked chromatin was recovered by centrifugation at 12,000 rpm for 10 min at 4 °C (the supernatant was discarded). FA-1 buffer (800 µl) was added to the top of the pellet. The chromatin was sonicated for 2 min (10 s ON, 15 s OFF, 20% amplitude) and then centrifuged for 15 min at 12,000 rpm at 4 °C; glycerol 5% was added to the supernatants. The sonicated chromatin was mixed with Sepharose Cl-4B beads (CL4B200; Sigma) and cleared for 1 h at 4 °C. Immunoprecipitation was performed by mixing “cleared-sonicated chromatin” with 35–40 mg IgG and anti-MYC antibody on a rotating wheel overnight at 4 °C. A 100-µl bed of Protein A Sepharose CL-4B beads (17-0780-01; GE Healthcare) was added and incubated for 2 h at 4 °C. The beads were recovered and washed successively with FA-1 buffer (plus CPI-EDTA 1×), FA-2 buffer (as FA-1 buffer but with 500 mM NaCl, plus CPI-EDTA 1×), FA-3 buffer (10 mM Tris-HCl at pH 8, 0.25 M LiCl, 0.5% NP-40, 0.5% w/v sodium deoxycholate, 1 mM EDTA at pH 8, plus CPI-EDTA 1×), and TE 1 × (100 mM Tris-Cl at pH 8, 10 mM EDTA at pH 8) at 4 °C. The cross-linking of the sonicated chromatin was reversed by incubating the washed beads with 250 µl TE buffer containing 1% SDS and 1 mg/ml proteinase K overnight at 65 °C. The DNA was purified using a Qiagen PCR purification kit and eluted with 55 µl of buffer EB containing RNase A (0.5 mg/ml).

### Quantitative real-time PCR

Amplification was performed in a 10-µl reaction with 5 µl 2× EvaGreen mix (MasterMix-S; Applied Biological Materials, Richmond, Canada), 0.8 µl each primer (10 nmol/litre), 2 µl sample complementary DNA, and 2.2 µl ddH2O. Real-time PCR was performed using a Roche Light Cycler 480II System (Roche Diagnostics, Mannheim, Germany). The PCR program was initiated at 95 °C for 10 min, followed by 40 cycles of denaturation for 5 s at 95°, annealing for 30 s at 60°, and elongation for 60 s at 72°. Fluorescence signals were observed at 72° during the elongation step. Each sample was analysed with at least three biological replicates and normalized to the IgG sample. The results were analysed using Light Cycle 480 SoftWare 1.5.1 in the Roche Light Cycler 480II System. All primers used in this study are described in Table S2.

### Meiotic surface spread nuclei

Meiotic chromosome spread, staining and imaging were carried out as previously described [61] with the following modifications: 80 µL 1xMES and 200 µL 4% paraformaldehyde were added to spheroplasted, washed cells, and then cells were lysed and spread on a glass microscope slide with 1% Lipsol (LIP Ltd., Shipley England) and fixed by 3% w/v paraformaldehyde with 3.4% w/v sucrose as described [61]. The slide was air dried until less than half of the liquid remained and then washed in 0.4% Photo-flo as described [61]. Primary antibodies (mouse monoclonal anti DNA:RNA hybrid [S9.6] (Kerafast, Cat # ENH001, 1:50), rabbit polyclonal anti-GFP (1:100, [62])) were added to the sections and incubated at 4 °C overnight, followed by incubation with the secondary antibodies. The nuclei were stained with DAPI. The images were taken immediately using an LSM 780 microscope (Zeiss, Oberkochen, Germany) or a TCS SP8 microscope (Leica, Wetzlar, Germany).

## Electronic supplementary material

Below is the link to the electronic supplementary material.

**Additional file 1:** Figures and Tables.

## Data Availability

The data generated during all experiments is available from the author upon reasonable request. The accession number for the ssDRIP data used in this paper is GSE167915.

## Abbreviations

AI: Anaphase I
ARS: Autonomously replicating sequences
cR-loops: Crick R-loops
DSBs: DNA double-strand breaks
MI: Metaphase I
MNase: Micrococcal nuclease
MRX: Mre11–Rad50–Xrs2
ssDNA: Single-stranded DNA
Pac: Pachytene stage
PCA: Principal component analysis
RNAPII: RNA polymerase II
ssDRIP-seq: Single-strand DNA ligation-based library construction from DNA:RNA hybrid immunoprecipitation, followed by sequencing
TES: Transcription end site
TFs: Transcription factors
TSS: Transcription start site
wR-loops: Watson R-loops
