# Supplementary material for: Dual roles of R-loops in the formation and processing of programmed DNA double-strand breaks during meiosis
Source: Cell Biosci. 2023 May 11;13:82. doi: 10.1186/s13578-023-01026-2 (PMC10173651; doi:10.1186/s13578-023-01026-2)
Supplement: Supplementary file 1 — Additional file 1: Figures and Tables. [file 13578_2023_1026_MOESM1_ESM.docx]

Supplementary Information

Additional file 1:

**Figure and Figure Legends**


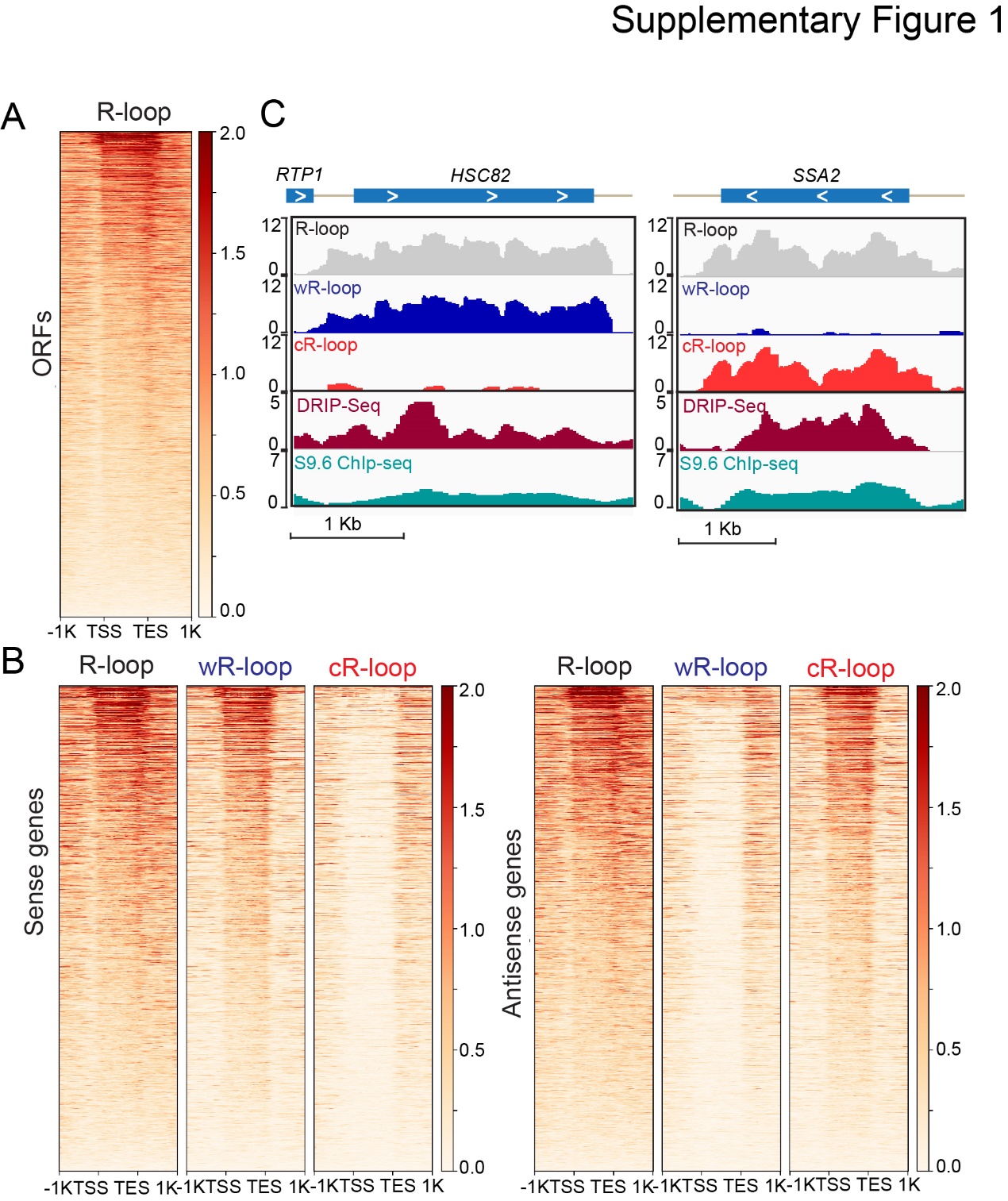


Figure S1. R-loops are highly associated with gene transcription.

(**A**) Heatmap of R-loop levels on Pol II transcripts. ORFs are aligned from the transcription start site (TSS) to the transcription end site (TES) and plotted ± 1 kb.

(**B**) Heatmap of R-loop, wR-loop and cR-loop peaks on Pol II transcripts with different orientations. The sense gene indicated that the transcript coding strands were Watson DNA strands. Antisense gene indicated that the transcript coding strands were Crick DNA strands. ORFs are aligned from the transcription start site (TSS) to the transcription end site (TES) and plotted ±1 kb.

(**C**) A representative genomic region covering the HSC82 and SSA2 gene loci, showing R-loop signals relative to the orientation of gene transcription. DRIP-seq data [1] and S9.6 ChIP-seq data [2] have been reported in wild-type yeast cells.


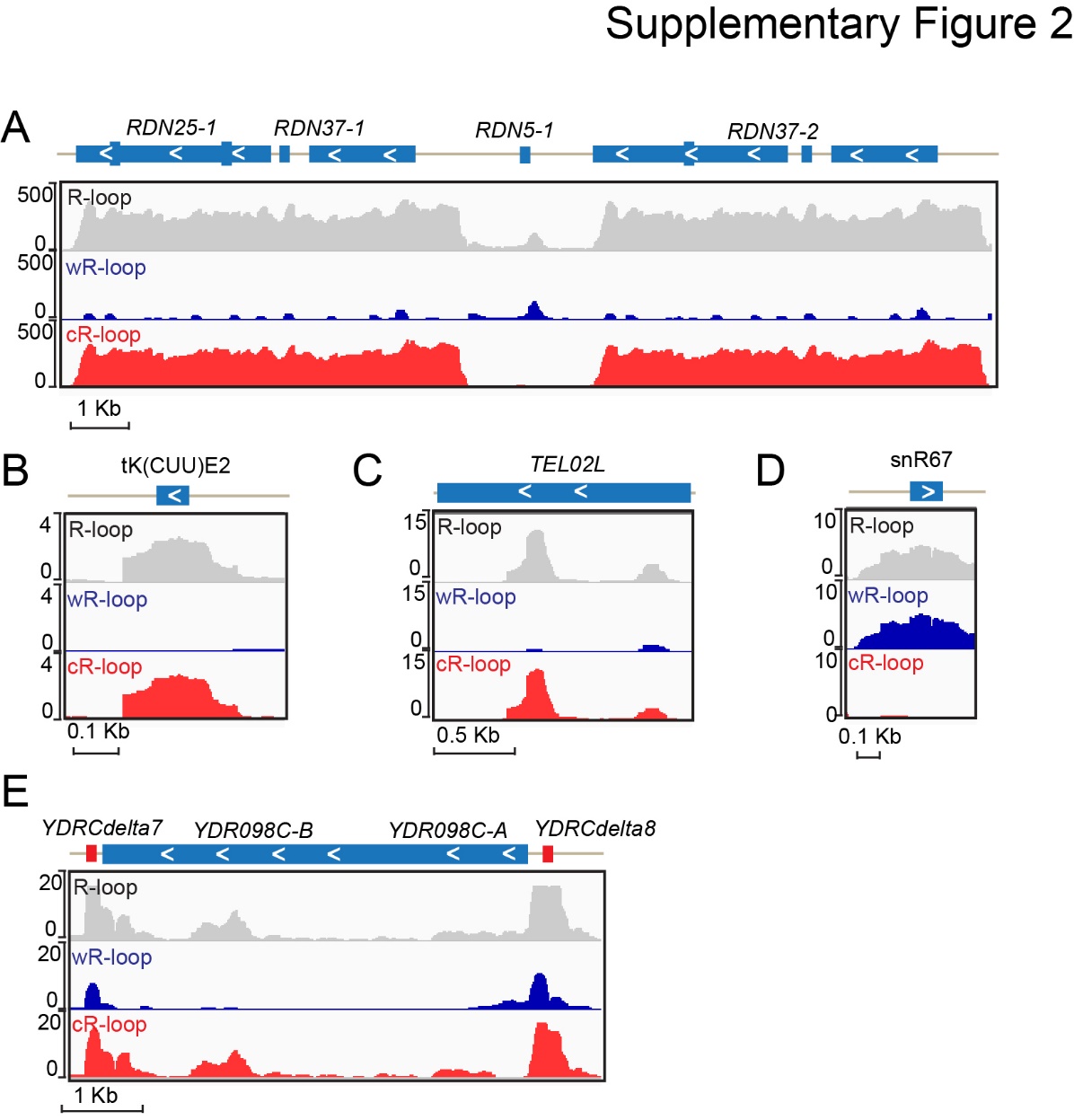


Figure S2. R-loops are strongly accumulated in rDNA, tRNA, telomeres, small nucleolar RNA and Ty elements.

Snapshots showing R-loops present in rDNA (A), tRNA [tK(CUU)E2] (B), telomeres [TEL02L] (C), small nucleolar RNA (snoRNA) [snR67] (D), and Ty elements [YDRCdelta7, YDRCdelta8] (E).


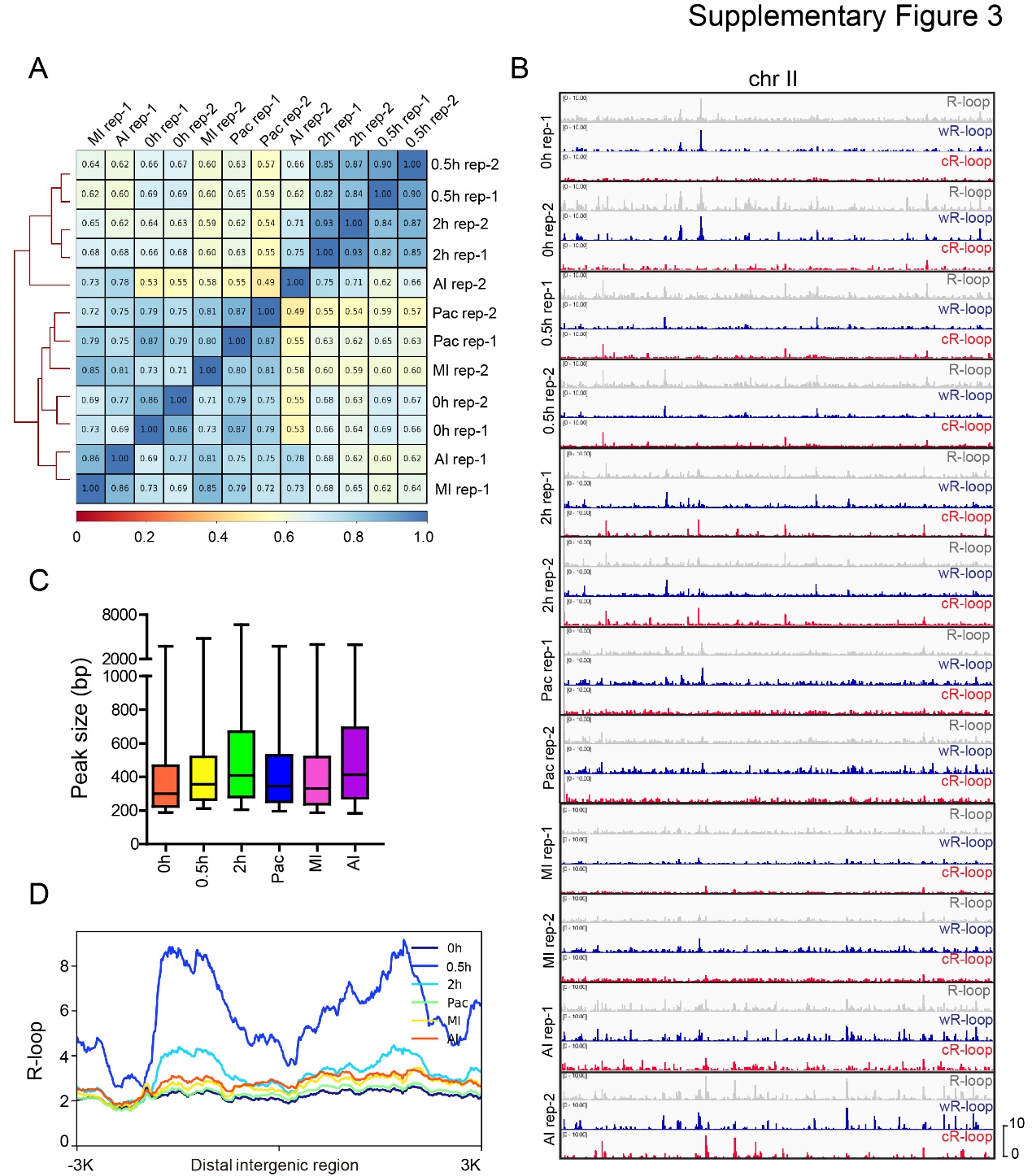


Figure S3. Genome-wide detection of R-loops during yeast meiosis.

(A) Pairwise comparison of two ssDRIP-seq replicates in meiosis. The Pearson correlation coefficient was computed from each comparison to evaluate the reproducibility.

(B) Snapshots showing R-loops on chr II during meiosis. The signal intensity in (B) is 0-10.

(C) The size distribution of R-loop peaks determined by the peak calling strategies of MACS2 during meiosis.

(D) Metaplots of R-loop peaks centred on distal intergenic regions (>500 bp from TSS and >300 bp from TES).


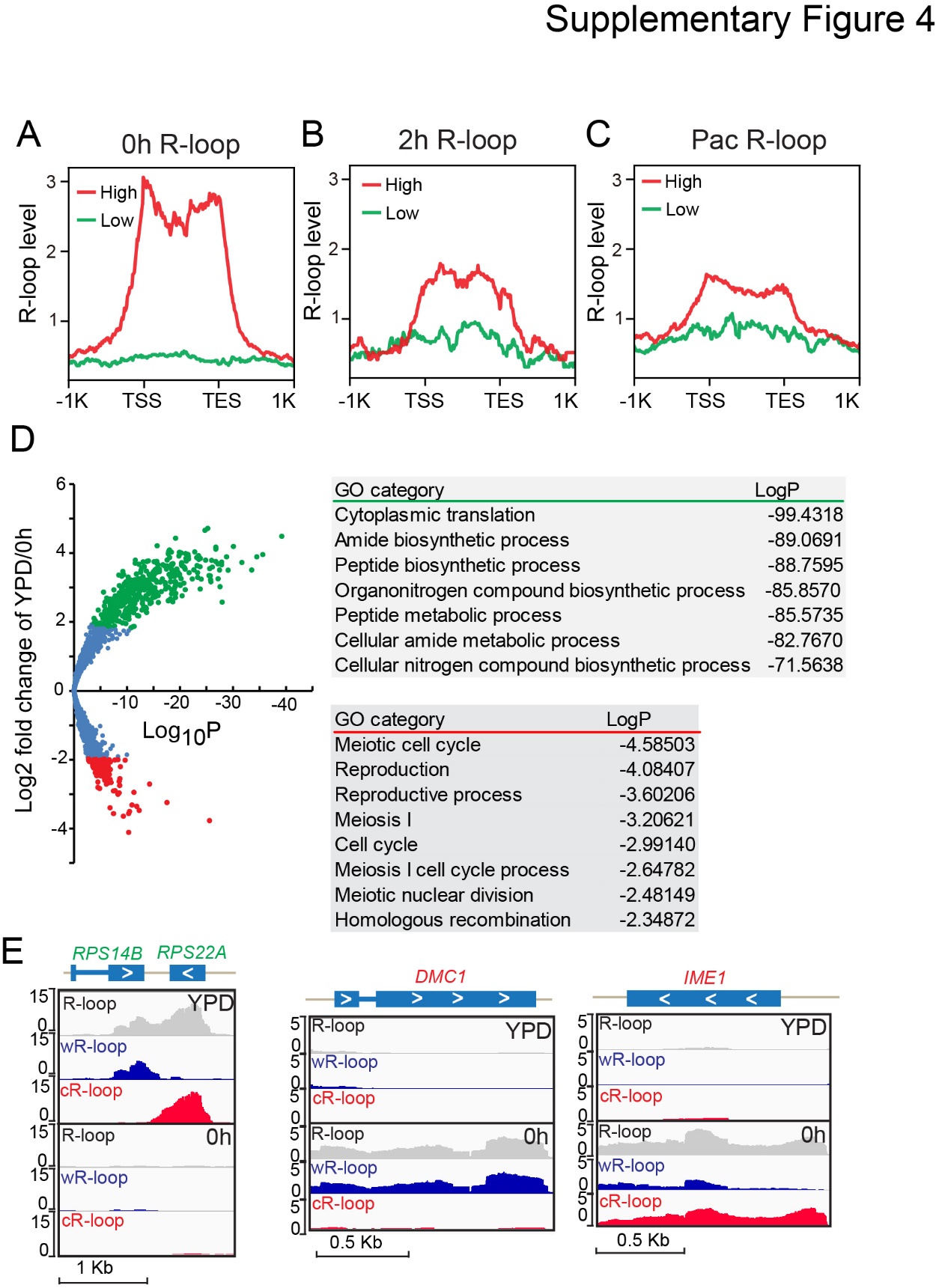


Figure S4. R-loops are highly associated with gene transcription during meiosis.

(**A**) Metaplots of the R-loop signal at 0 h after sporulation in regions ± 1 kb from the high and low expressed gene ORFs at the same time point from published RNA-seq data [3].

(**B**) Metaplots of the R-loop signal at 2 h after sporulation in regions ± 1 kb from the high and low expressed gene ORFs at the same time point from published RNA-seq data [3].

(C) Metaplots of the R-loop signal at the pachytene stage in regions ± 1 kb from the high and low expressed gene ORFs at the same time point from published RNA-seq data [3].

(D) R-loops from the vegetative growth stage (YPD) are compared with R-loops from the premeiotic entry stage (0 h) by DESeq2 algorithms. Functional classification analysis of up- and downregulated R-loop peak-associated genes was performed according to enrichment analysis of the biological process (BP).

(**E**) A representative genomic region covering the *RPS14B*, *RPS22A*, *DMC1* and *IME1* gene loci, showing R-loop signals relative to gene expression and the orientation of gene transcription.


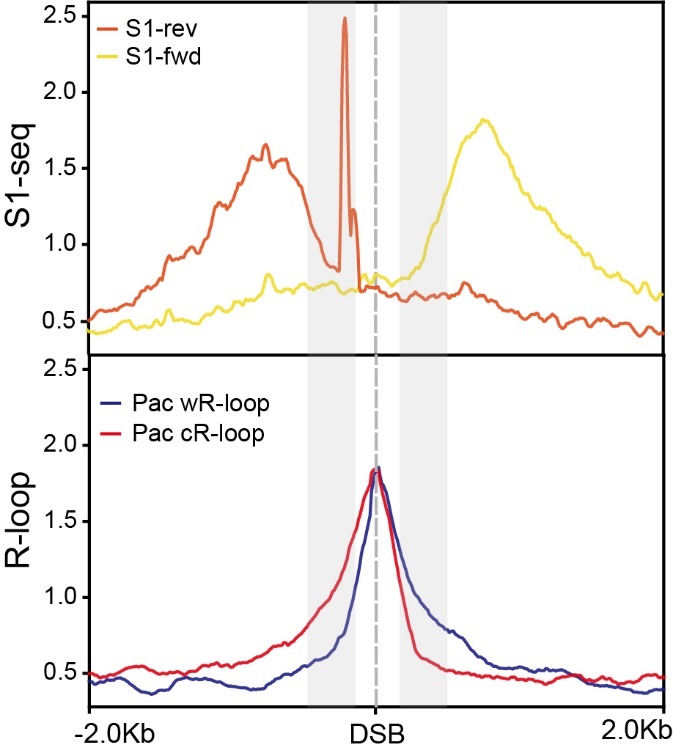


Figure S5. The wR-loop and cR-loop show a slight tendency to localize one side of the R-loop-associated hotspot centres. Metaplots of wR-loop and cR-loop peaks at the pachytene stage and S1-seq signals **[4]** in regions ± 2 kb from the centre of the colocalized Spo11-oligo-seq signal region in Figure 4B.


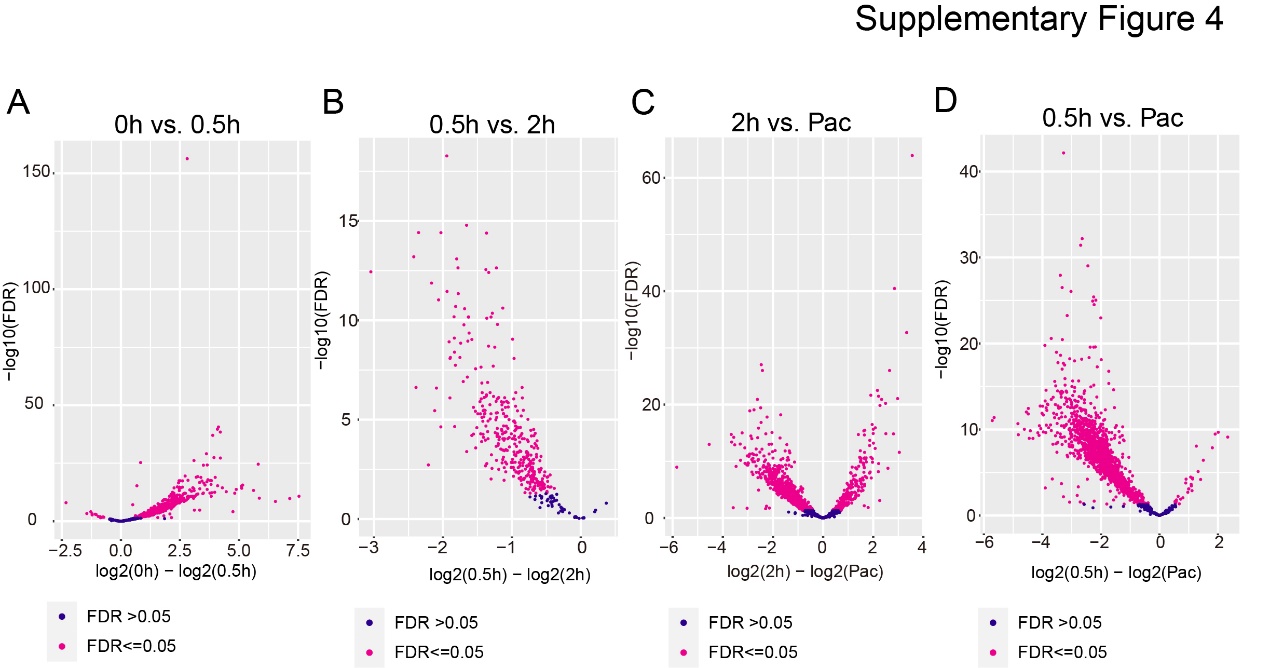


Figure S6. R-loop signals are dynamically changed at the early stage of meiosis.

(A) R-loops were decreased from 0 h to 0.5 h during yeast meiosis. FDR, False discovery rate.

(B) R-loops were increased from 0.5 h to 2 h during yeast meiosis. FDR, False discovery rate.

(C) R-loops were changed from the 2 h to pachytene stage during yeast meiosis. FDR, False discovery rate.

(D) R-loops were increased from 0.5 h to the pachytene stage during yeast meiosis. FDR, False discovery rate.


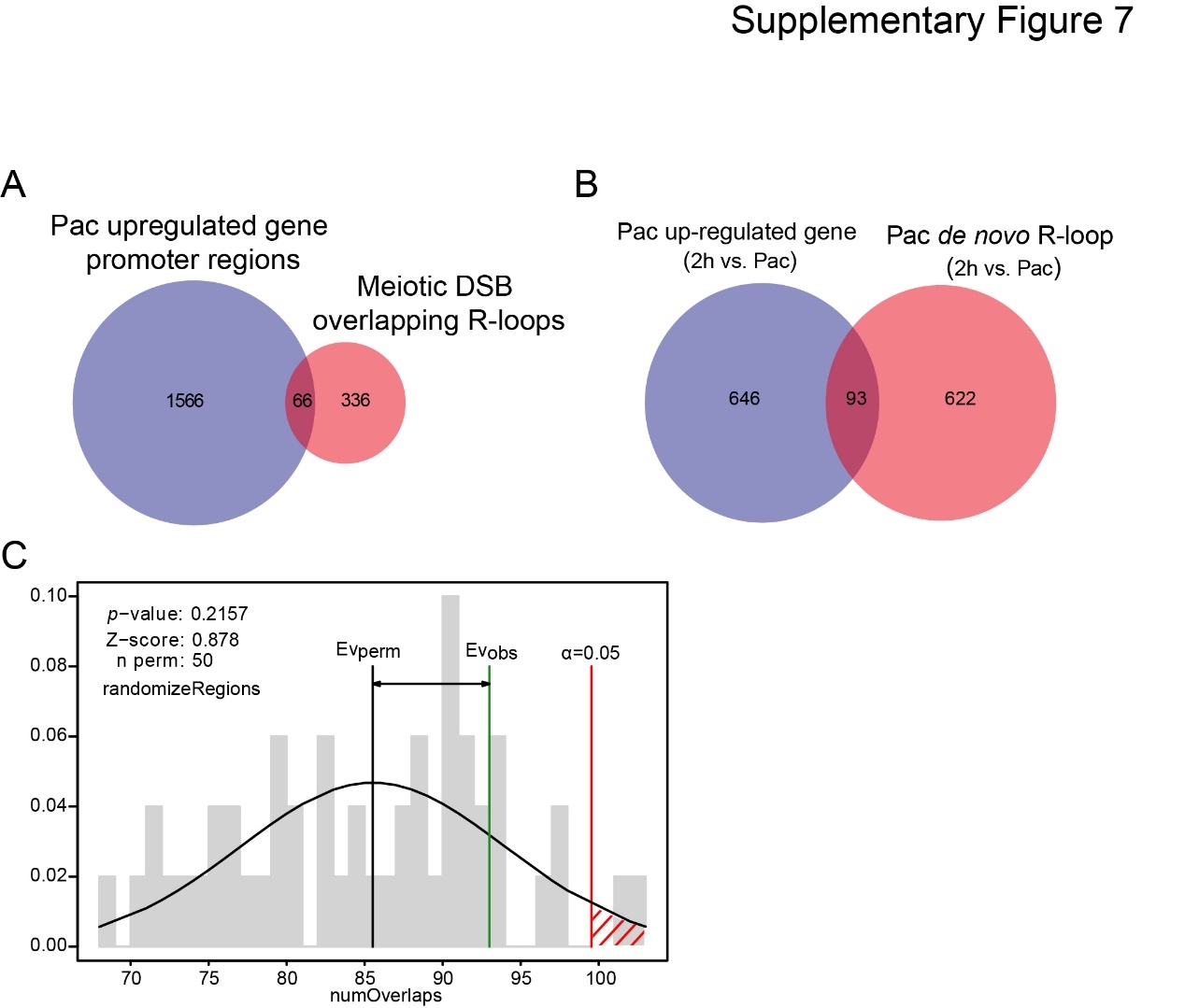


Figure S7. Correlation between the meiotic DSB hotspots and R-loops may not originate from highly expressed genes.

(A) Venn plots show the overlays of pachytene stage upregulated gene promoter regions [3] and meiotic DSB overlapping R-loops.

(**B**) Venn plots show the overlays of pachytene stage-upregulated genes (2 h vs. Pac) [3] and Pac *de novo* R-loops.

(C) Permutation test of colocalization between pachytene stage-upregulated genes (2 h vs. Pac) [3] and Pac *de novo* R-loops. Evobs, evaluation observe; Evperm, evaluation permutation.


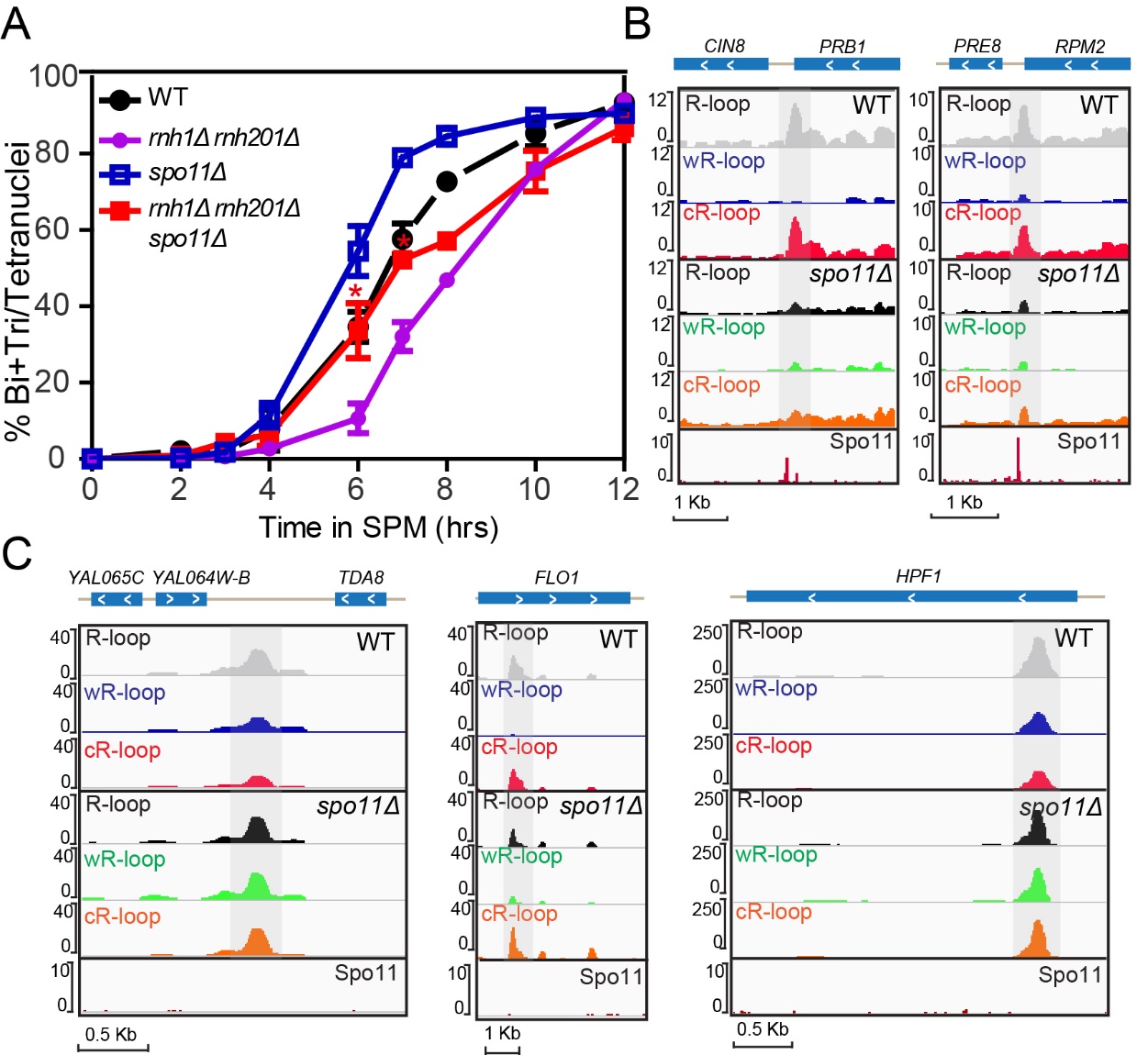


Figure S8. **Some R-loops are associated with Spo11 function during yeast meiosis.**

(A) Sporulation-related impacts resulting from deletion of the *SPO11*, *RNH1/RNH201* and *RNH1/RNH201/SPO11* loci. Data are presented as the means ± SEM. Two-tailed Student’s t test; **P* < 0.05.

(**B**) Representative genomic regions showing some R-loops co-localized with meiotic DSBs are decreased in *spo11Δ* strains.

(**C**) Representative genomic regions showing stronger R-loops signals out of meiotic DSBs are not affected by Spo11 depletion.


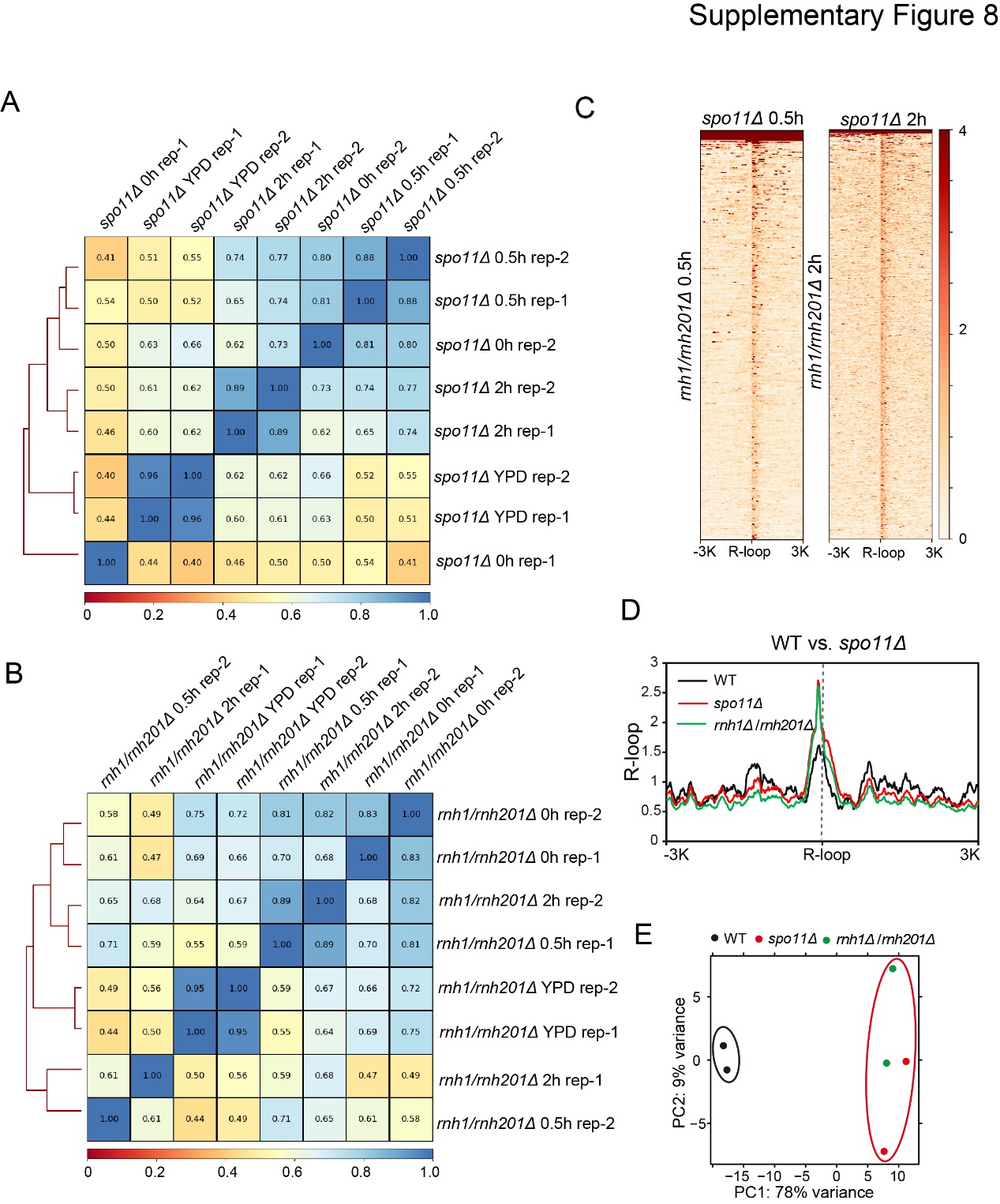


Figure S9. R-loop profile in ***spo11Δ*** and ***rnh1/rnh201Δ* cells** during mitosis and meiosis.

(A) Pairwise comparison of two ssDRIP-seq replicates in *spo11Δ* strains. The Pearson correlation coefficient was computed from each comparison to evaluate the reproducibility.

(B) Pairwise comparison of two ssDRIP-seq replicates in *rnh1/rnh201Δ* strains. The Pearson correlation coefficient was computed from each comparison to evaluate the reproducibility.

(**C**) Heatmap presentation of *spo11Δ* ssDRIP-seq signals in regions ±3 kb from R-loop centres in *rnh1/rnh201Δ*.

(D) The distribution of R-loops in the *rnh1*/*rnh201Δ* strain was similar to that of the *spo11Δ* strain. Metaplots of R-loop peaks centred on the accumulated R-loops after *SPO11* deletion after sporulation (WT vs. *spo11Δ*) in WT and *rnh1/rnh201Δ* strains.

(E) Principal component analysis (PCA) of R-loops in the WT, *spo11Δ* and *rnh1/rnh201Δ* strains at 2 h after sporulation.

Table S1**. Strains used in this study**

| **Strain** | **Genotype** | **Source** |
| --- | --- | --- |
| LW0066 | *MATa/α, ho::LYS2/ ho::LYS2, lys2/ lys2, ura3/ ura3, leu2::hisG/leu2::hisG, his3::hisG/ his3::hisG, trp1::hisG/ trp1::hisG* | [5] |
| A14201 | *MATa/a ura3::pGPD1-GAL4(848).ER::URA3/ura3::pGPD1-GAL4(848).ER::URA3 GAL-NDT80::TRP1/GAL-NDT80::TRP1* | [6] |
| LW2175 | *MATa/α, ho::LYS2/ ho::LYS2, lys2/ lys2, ura3/ ura3, leu2::hisG/leu2::hisG, his3::hisG/ his3::hisG, trp1::hisG/ trp1::hisG*  ***rnh1::HIS3/ rnh1:: HIS3*** | This study |
| LW2176 | *MATa/α, ho::LYS2/ ho::LYS2, lys2/ lys2, ura3/ ura3, leu2::hisG/leu2::hisG, his3::hisG/ his3::hisG, trp1::hisG/ trp1::hisG*  ***rnh201::KANMX4/rnh201::KANMX4*** | This study |
| LW2178 | *MATa/α, ho::LYS2/ ho::LYS2, lys2/ lys2, ura3/ ura3, leu2::hisG/leu2::hisG, his3::hisG/ his3::hisG, trp1::hisG/ trp1::hisG*  ***rnh1::HIS3/rnh1:: HIS3****;*  ***rnh201::KANMX4/rnh201::KANMX4*** | This study |
| LW2179 | *MATa/α, ho::LYS2/ ho::LYS2, lys2/ lys2, ura3/ ura3, leu2::hisG/leu2::hisG, his3::hisG/ his3::hisG, trp1::hisG/ trp1::hisG*  ***spo11::URA3/ spo11::URA3*** | This study |
| LW2180 | *MATa/α, ho::LYS2/ ho::LYS2, lys2/ lys2, ura3/ ura3, leu2::hisG/leu2::hisG, his3::hisG/ his3::hisG, trp1::hisG/ trp1::hisG*  ***spo11::URA3/ spo11::URA3;***  ***rnh1::HIS3/rnh1:: HIS3****;*  ***rnh201::KANMX4/rnh201::KANMX4*** | This study |
| LW2181 | *MATa/α, ho::LYS2/ ho::LYS2, lys2/ lys2, ura3/ ura3, leu2::hisG/leu2::hisG, trp1::hisG/ trp1::hisG*  ***his3::hisG/SPO11-9×MYC-HIS3*** | This study |
| LW2182 | *MATa/α, ho::LYS2/ ho::LYS2, lys2/ lys2, ura3/ ura3, leu2::hisG/leu2::hisG, trp1::hisG/ trp1::hisG*  ***his3::hisG/SPO11-9×MYC-HIS3***  ***ars315::KanMX4/ ars315::KanMX4*** | This study |
| LW2183 | *MATa/α, ho::LYS2/ ho::LYS2, lys2/ lys2, leu2::hisG/leu2::hisG, trp1::hisG/ trp1::hisG*  ***his3::hisG/SPO11-9×MYC-HIS3***  ***TAH1-rev-URA3/TAH1-rev-URA3*** | This study |
| LW2184 | *MATa/α, ho::LYS2/ ho::LYS2, lys2/ lys2, leu2::hisG/leu2::hisG, trp1::hisG/ trp1::hisG*  ***his3::hisG/SPO11-9×MYC-HIS3***  ***ARS306-TAH1-rev-URA3/ ARS306-TAH1-rev-URA3*** | This study |
| LW2185 | *MATa/α, ho::LYS2/ ho::LYS2, lys2/ lys2, ura3/ ura3, trp1::hisG/ trp1::hisG*  ***his3::hisG/SPO11-9×MYC-HIS3***  ***ars309::LEU2/ars309::LEU2*** | This study |
| LW2186 | *MATa/α, ho::LYS2/ ho::LYS2, lys2/ lys2, leu2::hisG/leu2::hisG, trp1::hisG/ trp1::hisG*  ***his3::hisG/SPO11-9×MYC-HIS3***  ***ADY2-rev-URA3/ADY2-rev-URA3*** | This study |
| LW2187 | *MATa/α, ho::LYS2/ ho::LYS2, lys2/ lys2, leu2::hisG/leu2::hisG, trp1::hisG/ trp1::hisG*  ***his3::hisG/SPO11-9×MYC-HIS3***  ***ARS319-ADY2-rev-URA3/ ARS319-ADY2-rev-URA3*** | This study |
| LW2188 | *MATa/α, ho::LYS2/ ho::LYS2, lys2/ lys2, ura3/ ura3, leu2::hisG/leu2::hisG, trp1::hisG/ trp1::hisG*  ***his3::hisG/SPO11-9×MYC-HIS3***  ***ars733::KanMX4/ ars733::KanMX4*** | This study |
| LW2189 | *MATa/α, ho::LYS2/ ho::LYS2, lys2/ lys2, leu2::hisG/leu2::hisG, trp1::hisG/ trp1::hisG*  ***his3::hisG/SPO11-9×MYC-HIS3***  ***MPC3-rev-URA3/ MPC3-rev-URA3*** | This study |
| LW2190 | *MATa/α, ho::LYS2/ ho::LYS2, lys2/ lys2, leu2::hisG/leu2::hisG, trp1::hisG/ trp1::hisG*  ***his3::hisG/SPO11-9×MYC-HIS3***  ***A******RS306-MPC3-rev-URA3/ARS306-MPC3-rev-URA3*** | This study |

Table S2. List of primer probe set sequences in this study.

| Name | **Sequence (5’ to 3’)** |
| --- | --- |
| ARS315-F | CTTGCATATTGCAATGTTCAC |
| ARS315-R | GCCGTGCTTCGTCGTAACTG |
| ARS309-F | TGCAGTCTTCCCCAACGTTTG |
| ARS309-R | CTGCAGCGAGATACGAAAAG |
| ARS733-F | CCCTTTTCCTCCTTGATTGG |
| ARS733-R | TACTCCAATTGCAACGCACC |

References

1. Garcia-Rubio M, Aguilera P, Lafuente-Barquero J, Ruiz JF, Simon MN, Geli V, Rondon AG, Aguilera A. Yra1-bound RNA-DNA hybrids cause orientation-independent transcription-replication collisions and telomere instability. Genes & Development. 2018; 32.965-977.

2. El Hage A, Webb S, Kerr A, Tollervey D. Genome-Wide Distribution of RNA-DNA Hybrids Identifies RNase H Targets in tRNA Genes, Retrotransposons and Mitochondria. Plos Genetics. 2014; 10.

3. Brar GA, Yassour M, Friedman N, Regev A, Ingolia NT, Weissman JS. High-resolution view of the yeast meiotic program revealed by ribosome profiling. Science. 2012; 335.552-557.

4. Mimitou EP, Yamada S, Keeney S. A global view of meiotic double-strand break end resection. Science. 2017; 355.40-45.

5. Liu C, Zhao H, Xiao S, Han T, Chen Y, Wang T, Ma Y, Gao H, Xie Z, Du LL, et al. Slx5p-Slx8p Promotes Accurate Chromosome Segregation by Mediating the Degradation of Synaptonemal Complex Components during Meiosis. Adv Sci (Weinh). 2020; 7.1900739.

6. Zhao H, Wang Q, Liu C, Shang Y, Wen F, Wang F, Liu W, Xiao W, Li W. A Role for the Respiratory Chain in Regulating Meiosis Initiation in Saccharomyces cerevisiae. Genetics. 2018; 208.1181-1194.
